# Cepharanthine inhibits enterovirus entry by endolysosomal deacidification and exhibits protective activity *in vivo*

**DOI:** 10.1128/aac.00764-25

**Published:** 2025-10-29

**Authors:** Ta-Chou Weng, Bang-Yan Hsu, Szu-Hao Kung

**Affiliations:** 1 Department of Biotechnology and Laboratory Science in Medicine, National Yang Ming Chiao Tung University, Taipei, Taiwan; Houston Methodist Hospital and Weill Cornell Medical College, Houston, Texas, USA

**Keywords:** enterovirus, antiviral drug, bisbenzylisoquinoline alkaloids, cepharanthine, viral entry, lysosome

## Abstract

Enteroviruses, which belong to the *Picornaviridae* family, are linked to a range of clinical illnesses that vary from mild to severe, including life-threatening diseases. Among these, enterovirus 71 (EV71) infections in infants and young children can lead to serious neurological conditions, posing a significant public health risk due to the absence of approved treatments. In this study, we assessed the anti-EV activities of four bisbenzylisoquinoline alkaloids (BBAs): tetrandrine (TET), cepharanthine (CEP), fangchinoline (FAN), and berbamine (BER), as well as their mechanisms of action. In all cases, we observed a dose-dependent decrease in EV71 protein levels and viral titers. TET and CEP exhibited lower 50% inhibitory concentrations and higher selectivity indexes among the tested BBAs. Therefore, we prioritized TET and CEP for mechanistic investigation and *in vivo* evaluation. Mechanistic studies revealed that TET and CEP inhibited EV71 infection primarily at the entry stage, without impacting viral binding, internalization, or post-entry processes. Further studies demonstrated that TET and CEP disrupted viral trafficking along the endolysosomal pathway. Both compounds were found to effectively neutralize low pH levels in endolysosomes, which corresponded to the reduced antiviral effects caused by the acidic replenishment of the medium. The antiviral effects of TET and CEP were observed against various serotypes of EV. Remarkably, administering CEP at a dose of 10 mg/kg provided complete protection to mice infected with EV71 from lethal challenges, significantly reducing viral titers, viral RNA levels, and pathological scores. Collectively, these findings highlight CEP as a promising candidate for the treatment of EV infections.

## INTRODUCTION

Enteroviruses are part of the *Picornaviridae* family and are characterized as non-enveloped, positive-sense RNA viruses. The Enterovirus (EV) genus includes several significant human pathogens, such as polioviruses, coxsackieviruses types A and B, echoviruses, numbered enteroviruses, and rhinoviruses. While most EV infections result in mild or asymptomatic illnesses, they can also lead to serious and potentially life-threatening conditions, including myocarditis, pancreatitis, meningitis, encephalitis, and acute paralysis (1, 2). Among these viruses, EV71 (or EV-A71) is a significant cause of hand, foot, and mouth disease (HFMD), particularly affecting young children and infants. A notable proportion of those infected with EV71 may experience severe neurological complications, and in some cases, this can lead to death, especially in the Asia-Pacific region (3). Currently, vaccines are available for poliovirus and EV71 infections (4, 5); however, developing vaccines for all enteroviruses is challenging due to the wide variety of serotypes. Additionally, there are no approved antiviral treatments for EV infections at this time, highlighting the urgent need for broad-acting antiviral medications to address the diverse and potentially severe effects of EVs.

The life cycle of all EVs begins with their specific binding to one or more cell surface receptors, initiating the process of receptor-mediated endocytosis (6–8). After a virus attaches to its receptor, changes in pH within the endosome facilitate virus trafficking, resulting in the release of the viral genome into the cytoplasm (6, 9, 10). Upon entering the cytosol, the positive-stranded viral genome acts as an mRNA and is translated into a polyprotein. This translation utilizes an internal ribosome entry site (IRES) within the 5′ untranslated region of the viral genome, allowing for efficient, cap-independent translation. The resulting polyprotein is then effectively cleaved by virus-encoded proteases, specifically 2A and 3C, generating vital structural proteins known as VP1-4, along with essential non-structural proteins, including proteases and polymerases. The replication of the viral genome is propelled by the viral RNA-dependent RNA polymerase (3D). The newly formed positive-sense progeny viral RNAs are meticulously packaged into capsids to create new virions, which are subsequently released from host cells through either lytic or non-lytic mechanisms (11, 12).

Bisbenzylisoquinoline alkaloids (BBAs) are a class of natural products derived from plants of *Berberidaceae*, *Monimiaceae,* and *Ranunculaceae* families. These compounds are formed by linking two benzyl isoquinoline units through one, two, or three ether linkages.

BBAs are known for their diverse biological activities, which include anti-inflammatory effects (13), antioxidant properties (14), and anti-tumor responses (15), as well as anti-infective actions against bacteria (16), parasites (17), and viruses. Research has shown that BBAs, including tetrandrine (TET), cepharanthine (CEP), fangchinoline (FAN), and berbamine (BER), specifically target different stages of enveloped viral infections. For instance, TET disrupts the transport of viruses like Ebola, severe acute respiratory syndrome coronavirus 2 (SARS-CoV-2), and the African swine fever virus (ASFV) from early endosomes to late endosomes (18–20). Similarly, CEP inhibits the entry phase of hantavirus and SARS-CoV-2 (21–23) , while also affecting the post-entry phases of herpes simplex virus 1 (HSV-1) and hepatitis B virus (HBV) (24, 25). Additionally, CEP can simultaneously block both entry and post-entry replication of the HIV through distinct mechanisms (26, 27). On the other hand, FAN inhibits the replication of the porcine epidemic diarrhea virus by obstructing autophagic flux (28). Meanwhile, BER has been shown to prevent Japanese encephalitis virus (JEV) and SARS-CoV-2 infections by interfering with the endolysosomal trafficking of viral receptors, such as the low-density lipoprotein receptor and angiotensin-converting enzyme 2, respectively (29).

The antiviral activities of BBAs are primarily effective against enveloped viruses; nevertheless, a recent report indicated that FAN inhibits several serotypes of EV in cell cultures (30). In this study, we aimed to evaluate the antiviral potency of TET, CEP, FAN, and BER, as well as to explore the underlying mechanisms of action. Our findings revealed that CEP exhibited the most potent antiviral activity among the tested BBAs, demonstrated by the lowest 50% inhibition concentrations (IC_50_) value and the highest selectivity index (SI). We focused on CEP and showed that it likely inhibits EV replication by neutralizing the low-pH endolysosomal pathway and interfering with the virus endolysosomal trafficking, resulting in blocking the viral entry. Notably, CEP displayed broad-spectrum anti-EV effects. Moreover, we demonstrated that CEP administration significantly reduced the lethal effects of EV71 infection and lowered the viral levels in a mouse model, suggesting its potential development as a novel antiviral treatment for EV infections.

## MATERIALS AND METHODS

### Cells and viruses

HeLa cells (ATCC, CCL-2) and Rhabdomyosarcoma (RD) (ATCC, CCL-13) cells were cultured at 37°C in minimum essential medium (MEM) (Gibco-BRL, Inc.) supplemented with 10% fetal bovine serum (31). EV stocks used in this research included EV71 (BrCr strain), coxsackievirus A16 (CVA16), CVB1, CVB3, echovirus serotype 9 (Echo9), Echo30, and EV68 (Fermon strain), as documented (32). A mouse-adapted EV71 MP4 strain used for the mouse model was kindly provided by Dr. Jen-Ren Wang from National Cheng Kung University, Taiwan.

### Chemicals

Tetrandrine (518-34-3), cepharanthine (481-49-2), fangchinoline (436-77-1), berbamine (6078-17-7), and emetine (7083-71-8) were purchased from Cayman Chemical. Chloroquine (C6628-25G) was purchased from Sigma. All compounds were dissolved in dimethyl sulfoxide (DMSO), and the final DMSO concentration in the culture medium did not exceed 0.05%, a concentration tolerated by all tested cell lines. All drug-free controls contained 0.05% DMSO.

### Virus infection and titration

Virus infections were conducted in MEM supplemented with 2% FBS at 37°C unless otherwise indicated. The supernatant containing extracellular viruses was collected from the EV71-infected cell cultures and centrifugated at 5,700 × *g* for 5 min to remove cell debris. Cell-associated viruses were prepared from cell lysates collected after freeze-and-thaw cycles and centrifugation at 15,300 × *g* for 10 min. The total virus is the combined mixture of supernatant and cell-associated virus prepared from above. Infectious viral titer was measured using the 50% tissue culture infectious dose (TCID_50_) method on RD cells by the method of Reed-Muench (33).

### Western blot

A western blot analysis was conducted as previously described (31). The primary antibodies (Ab) were a Mouse EV71 monoclonal VP1 Ab (1:3,000, GTX633390, GeneTex) and a rabbit anti-α tubulin primary Ab (1:10,000, GTX112141, GeneTex), the latter serving as the loading control. Secondary Abs included an HRP-conjugated goat anti-mouse polyclonal Ab (1:1,000, sc-2030, Santa Cruz) and a goat anti-rabbit HRP-conjugated secondary Ab (1:3,000, sc-2004, Santa Cruz). Proteins were detected using an enhanced chemiluminescence western blot kit (GTX14698, GeneTex). The band intensities were quantified using ImageJ software.

### Immunofluorescence assay

HeLa cells were seeded in 24-well plates and pre-treated with various concentration of test compounds for 1 h. After the culture media was removed, the cells were subsequently infected with EV71 at a multiplicity of infection (MOI) of 0.1 for 1 h. Following adsorption, the inoculum was discarded, and the cells were washed twice with phosphate-buffered saline (PBS). Medium containing the same concentration of the compound was then added. At 12 h post-infection (p.i.), cells were fixed with 4% paraformaldehyde and permeabilized using 0.2% Triton X-100. Mouse anti-EV71 monoclonal Ab (1:2,000, MAB979, Millipore) and FITC-conjugated goat anti-mouse Ab (1:200, 115-095-062, Jackson ImmunoResearch) were used as a primary Ab and secondary Ab, respectively. The nuclei were stained with DAPI-Aqueous (ab104139, Abcam). The cells were viewed under a fluorescent microscope (Leica DM6000B) equipped with both fluorescein isothiocyanate (FITC) and UV filters. EV antigen-positive cells and 4′,6-diamidino-2-phenylindole (DAPI) positive cells from each field were counted and analyzed using the associated MetaMorph software.

### Cell viability assay

Compound concentrations at 1, 3, 10, 30, and 100 µM were tested. Cell viability was assessed after 18 h of treatment using the CellTiter 96 AQueous Cell Proliferation Assay (Promega), as described (32). The 50% cytotoxic concentration (CC_50_) was determined using GraphPad Prism 9 (GraphPad Software).

### RNA extraction and reverse transcriptase quantitative PCR

RNA preparation and RT-qPCR followed the protocol previously detailed (31). Total cellular and viral RNA was extracted using a TRIzol reagent (ThermoFisher). The reverse transcription (RT) reaction and real-time PCR were performed using an AMV Reverse Transcriptase XL (Takara) and the FastStart Universal SYBR Green Master kit (Roche Applied Science), respectively, according to the manufacturer’s instructions. PCR primer pairs for the VP1 region of the EV71 (BrCr strain) genome and human β-actin were reported.

### Binding and internalization assay

HeLa cells were pre-treated with 5- and -10 µM of TET and CEP at 37°C for 2 h. After this incubation, the cells were placed at 4°C and incubated for 10 min in 1 mL of binding buffer composed of PBS, 1% bovine serum albumin (BSA), and 0.1% sodium azide. Following the binding buffer incubation, the cells were infected with EV71 at an MOI of 50 and kept at 4°C for 1 h to facilitate viral adsorption. Unbound virus was then washed off using PBS at 4°C, and the cells were treated with 0.25% trypsin (Gibco) to detach any virus bound to the cell surface. For the internalization assay, the unbound virus was removed after the binding period and replaced with pre-warmed medium containing the respective concentrations of the compounds. The cells were then incubated at 37°C for 1 h. To remove any surface-bound viruses, the cells were trypsinized at 37°C for 3 min. Finally, viral RNA was quantified using RT-qPCR to assess the viral level.

### EV71 replicon assay

The pSVA-EV71-GFP plasmid (6), a sub-genomic replicon of EV71 that encodes green fluorescent protein (GFP) reporter (a kind gift from Dr. Lih-Hwa Hwang, National Yang-Ming University), was linearized using the Not I restriction enzyme and subsequently utilized for *in vitro* transcription with the RiboMAX Large Scale RNA Production System-T7 (Promega, P1320). Following transcription, the RNA was precipitated by LiCl (Cat. #7447-41-8, Ambion AM9480) and washed with ice-cold 70% ethanol. HeLa cells were transfected with the pSVA-EV71-GFP RNA using Lipofectamine 2000 (ThermoFisher) according to the manufacturer’s instructions. After 30 min, the transfection reagent was removed and replaced with a medium containing the TET or CEP at 5 and 10 µM. The cells were incubated for 6 h, after which they were fixed with 4% paraformaldehyde and permeabilized with 0.2% Triton X-100. Nuclei were stained using an aqueous DAPI-containing mounting medium. The cells were then observed under a fluorescent microscope (Leica DM6000B) equipped with both FITC and UV filters. GFP-positive cells and DAPI-stained nuclei were counted and analyzed using MetaMorph software.

### EV71 movement analysis

HeLa cells were seeded in 24-well plates and incubated with EV71 at an MOI of 300 at 4°C for 1 h. After incubation, the cells were washed three times with PBS and then treated with either compound-containing or compound-free media for intervals of 15, 30, 45, 60, and 90 min. Following this, the cells were fixed with 4% paraformaldehyde and permeabilized using 0.2% Triton X-100. To detect the viral antigen, we used rabbit anti-EV71 VP2 polyclonal Ab (1:2,000, GTX132340, GeneTex) as the primary Ab and Goat Anti-Rabbit IgG H&L (Alexa Fluor 488) (1:500, ab150077, Abcam) as the secondary Ab. Additionally, cells were co-stained with either EEA1 Ab Alexa Fluor 546 (G-4, 1:500, sc-137130, Santa Cruz) or LAMP1 Ab Alexa Fluor 546 (H4A3, 1:500, sc-20011, Santa Cruz). The cells were also stained with DAPI to indicate the location of the nucleus. The stained samples were then examined using a confocal laser scanning microscope (Carl Zeiss LSM 880 META). Green fluorescence represented the viral antigen, red fluorescence corresponded to the early endosome antigen (EEA1) or lysosomal-associated membrane protein 1 (LAMP1), and blue fluorescence marked the nucleus. The percentage of colocalization was calculated using ZEN 2 software.

### Acidic organelle staining

Cells were cultured in 24-well plates and incubated with TET and CEP at the indicated concentrations at 37°C for 2 h. Lysotracker DND-99 (ThermoFisher, L7528) was used to stain acidic organelles within the cells. After removing the compound-containing medium, the cells were replaced with fresh medium containing the same compound and 500 nM Lysotracker DND-99, followed by an additional incubation at 37°C for 1 h. The cells were then washed three times with PBS, fixed with 4% paraformaldehyde, and permeabilized with 0.2% Triton X-100. Nuclei were stained using an aqueous DAPI-containing mounting medium. Fluorescence images were acquired on a Leica DM6000B microscope equipped with a red filter set (excitation 545 ± 30 nm; emission 610 ± 75 nm) specific for LysoTracker DND-99. Fluorescence intensity was quantified using ImageJ software.

### Low-pH exposure assay

We followed a protocol reported in a previous study with some modifications (31). HeLa cells were pre-treated for 1  h at 37°C with 10  µM TET or 8  µM CEP for 1 h at 37°C. After pretreatment, the cells were infected with EV71 at an MOI of 0.5 in the presence of 10 µM of either TET or CEP at 4°C. The inoculum was then removed, and unbound virus was washed away with ice-cold PBS. The cells were incubated for 1 h at 37°C in the presence of the respective compound. Afterward, the supernatant was discarded, and media with different pH levels (7.4, 6.5, 5.5, and 5.0), along with the corresponding compound, were added for 10 min. Finally, the media were removed, and the cells were incubated in a medium (pH 7.4) containing the respective compound at 37°C for 6 h.

### Mouse infection and sample analysis

The mouse study followed a protocol with some modifications (34). Seven-day-old ICR mice were infected with the MP4 virus via intraperitoneal injection at a dose of 1 × 10^7^ TCID_50_.

TET was administered at a dose of 10 mg/kg, while CEP was given at either 5 mg/kg or 10 mg/kg, with all treatments dissolved in DMSO. DMSO alone was also used as a control. The treatment was administered intraperitoneally 12 h prior to infection and continued every 12 h for four additional doses following the viral inoculation. The mice were monitored for body weight, survival rate, and clinical symptoms over a period of 7 days. Clinical severity was scored as follows: 0—healthy; 1—ruffled fur and hunchback appearance; 2—wasting; 3—limb weakness; 4—limb paralysis; and 5—moribund or death. Five mice in each group were euthanized at 7 days p.i. Their brain, spinal cord, and hind-limb muscle tissues were collected for viral titration and assessment of viral RNA levels using the TCID_50_ assay and RT-qPCR, respectively. Additionally, hematoxylin-and-eosin (H&E) staining was performed to evaluate histopathology. The lesion areas were quantified using ImageJ software, calculating the percentage of brain vacuole regions or myositis/myonecrosis regions in relation to the total tissue area within each field of view, as referenced in 35, 36.

## RESULTS

### BBAs effectively inhibit EV71 infections with CEP showing superior potency

**Fig 1:**
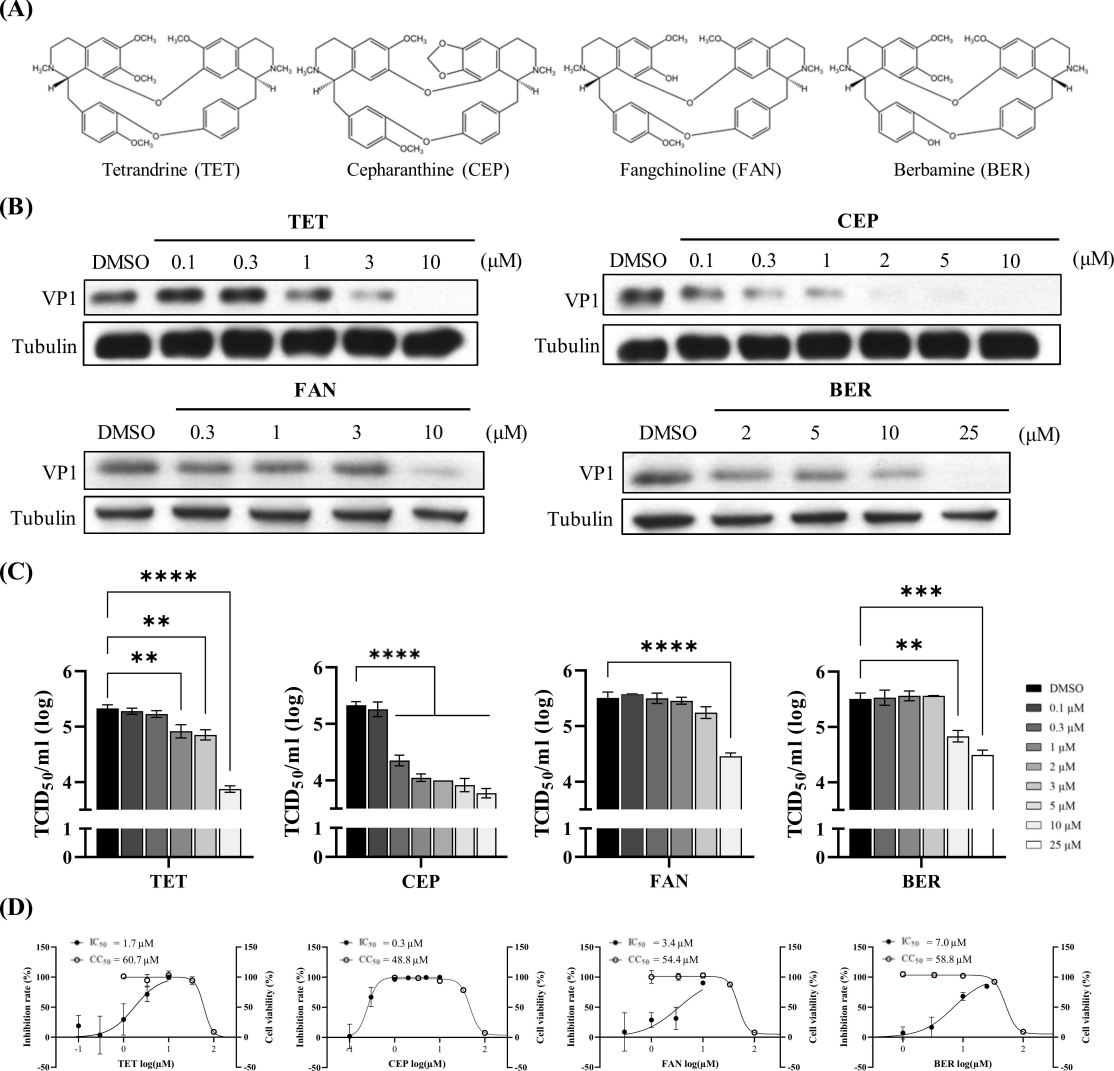
BBAs inhibit EV71 infection in a dose-dependent manner. (**A**) Chemical structures of CEP, TET, FAN, and BER. (**B**) HeLa cells were pretreated with TET, CEP, FAN, or BER at the indicated doses for 1 h, followed by infection with EV71 at an MOI of 0.5, with the corresponding compounds present for an additional 6 h. Cell lysates were prepared and subjected to Western blot analysis using an anti-EV71 VP1 Ab and an anti-tubulin Ab as an internal control. (**C**) HeLa cells were pretreated with TET, CEP, FAN, or BER at the indicated doses for 1 h, followed by infection with EV71 at an MOI of 0.5 for 8 h. Cell lysates and supernatants were collected, and total viral titers were determined by the TCID₅₀ assay. Data represent the mean ± standard deviation (SD) of three independent experiments performed in duplicate (*n* = 2). Statistical analysis was performed using one-way ANOVA; ****P < 0.0001, ***P < 0.001, **P < 0.01, *P < 0.05. (**D**) HeLa cells were pretreated for 1 h at the indicated concentrations of the test compounds and then infected with EV71 stocks at an MOI of 0.1, with test compounds maintained throughout the infection. At 12 p.i., the cells were analyzed using an IFA. For each condition, the percentage of infection was calculated as the ratio of the number of infected cells stained for viral VP1 to the number of cells stained with DAPI. Drug cytotoxicity was determined by treating HeLa cells with increasing concentrations of the indicated drugs (1, 3, 10, 30, and 100 µM) for 18 h. Cell viability was measured using a cell proliferation assay and expressed as the percentage of drug-free cells. IC_50_ and CC_50_ values were calculated using GraphPad Prism9 software. The solid circle and empty circle represented the inhibition rate (%) and cell viability (%), respectively. Data represented the means of triplicated experiments and the standard error of the mean (SEM). Drug-free wells contained 0.05% DMSO.

The chemical structures of BBAs, including TET, CEP, FAN, and BER, consist of two benzyl isoquinoline units linked together by oxygen bridges (Fig. 1A). We evaluated the anti-EV71 potency of these BBAs using Western blot analysis at specified concentrations in HeLa cells. In all cases, we observed a dose-dependent inhibition of viral protein (Fig. 1B). Moreover, we measured viral titers and protein levels in response to BBA treatments using the TCID_50_ assay (Fig. 1C) and an IFA (Fig. 1D; Fig. S1), respectively, and the IC_50_ values were determined using both methods (Table 1). Additionally, a cell viability assay was performed to determine the CC_50_ for each compound, along with calculating the SI, defined as SI = CC_50_/IC_50_. The results showed that the antiviral potencies of the compounds were ranked as follows: CEP > TET > FAN > BER, regardless of the methods employed. Notably, CEP exhibited low sub-micromolar IC_50_ values, with SI values reaching as high as 287 (by TCID_50_ assay) or 163 (by IFA).

**TABLE 1:** Antiviral potency and the selectivity indexes of BBAs against EV71

Compound	IC_50_ (µM)^*a*^		SI^*d*^		CC_50_ (µM)^*e*^
	TCID_50_^*b*^	IFA^*c*^	TCID_50_	IFA	
TET	0.9 ± 0.45	1.7 ± 0.86	67	36	60.7 ± 8.9
CEP	0.17 ± 0.08	0.3 ± 0.04	287	163	48.8 ± 4.7
FAN	3.2 ± 0.67	3.4 ± 1.05	17	16	54.4 ± 8.8
BER	8.9 ± 2.00	7.0 ± 1.70	7	8	58.8 ± 6.1

^*a*^ 50% inhibitory concentration.

^*b*^ 50% tissue culture infective dose.

^*c*^ Immunofluorescence assay.

^*d*^ Selectivity index, the ratio of CC_50_ to IC_50_.

^*e*^ 50% cytotoxic concentration.

### Time-of-addition analysis of TET and CEP

**Fig 2:**
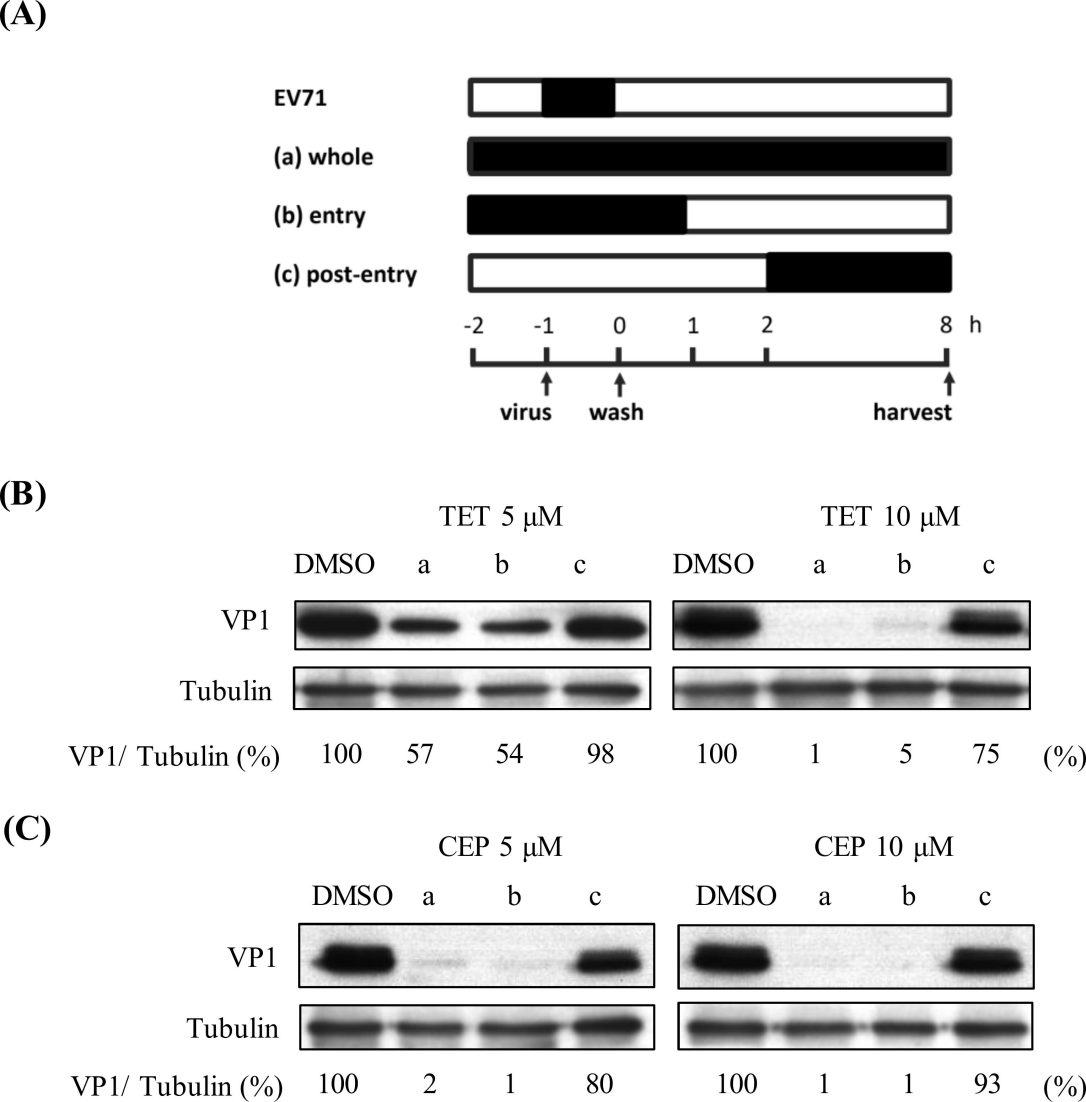
Timing of EV71 inhibition by TET and CEP. (**A**) Schematic timeline of drug addition illustrating the intervention of compounds at different stages of the EV71 life cycle during the entire infection period (a), during the viral entry phase (b), or during the post-entry phase (c). The initiation (−1) and completion (0) of viral inoculation are indicated. Filled and empty boxes represent the periods with and without compound treatment, respectively. HeLa cells were infected with EV71 at an MOI of 0.5 in the presence of TET (**B**) or CEP (**C**) at concentrations of 5 and 10 µM. Cell lysates were prepared for Western blot analysis using anti-VP1 and anti-tubulin Abs. The percentage shown below each lane represents the intensity of viral VP1 relative to that of tubulin.

We next focused on the two most effective drugs: TET and CEP. To better understand how these compounds affect the viral replication cycle, we conducted a time-of-addition assay (Fig. 2A). TET and CEP were administered to HeLa cells at concentrations of 5 or 10 µM during different phases of the infection process: (a) 1 h before and during the 1 h virus adsorption step, with treatment maintained throughout the subsequent 8 h infection period; (b) 1 h before and during the 1 h virus adsorption step, followed by an additional 1 h of treatment before removal (entry step); or (c) 2 h after the completion of adsorption step, continuing treatment for the remaining 6 h of infection (post-entry). For each condition, we analyzed viral protein levels using Western blotting. Our results demonstrated that treatment with TET (Fig. 2B) or CEP (Fig. 2C) primarily reduced viral protein levels during the entry phase, with minimal effects observed during the post-entry phase.

### TET and CEP do not impede the attachment, internalization, or post-entry processes of EV71

**Fig 3:**
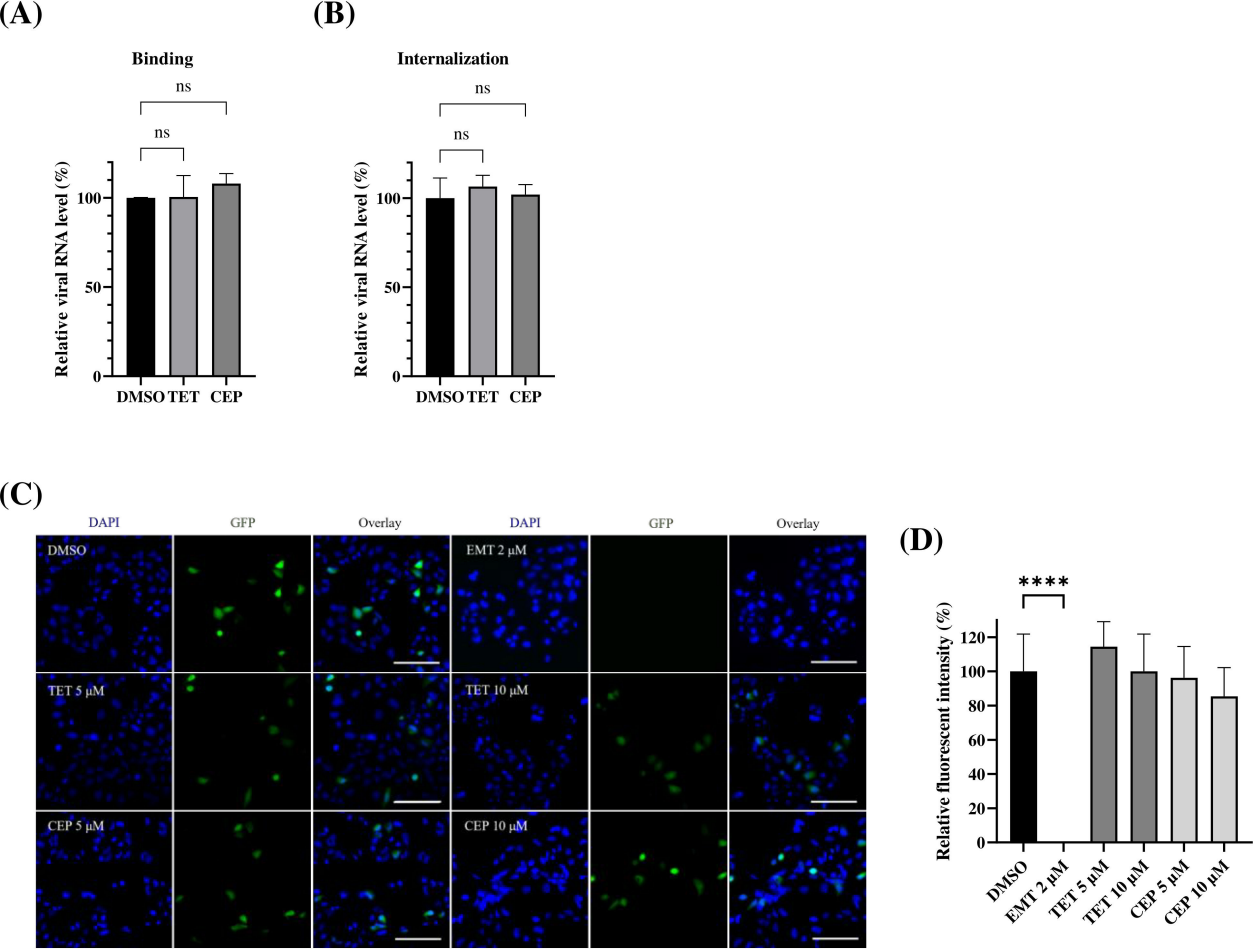
Antiviral activities of TET and CEP are independent of viral binding, internalization or the post-entry stage of viral infection. (**A, B**) HeLa cells were pretreated with 10 µM of the compound at 37°C for 2 h. The cells were then placed at 4°C and treated with binding buffer for 10 min. Following this, 50 MOI of EV71 was added and incubated at 4°C for 1 h to allow the virus to adsorb onto the cells. For the binding assay, unbound virus was washed off with PBS at 4°C, and the cells were treated with trypsin to detach any virus bound to the cell surface. For the internalization assay, after the initial virus adsorption at 4°C, cells were incubated at 37°C for an additional 1 h to allow for viral internalization. Cells were then washed with PBS and treated with trypsin to eliminate any remaining virus on the cell surface. Finally, the viral load from both assays was quantified using RT-qPCR. RNA level was normalized with β-actin as internal control. (**C**) HeLa cells were transfected with RNAs derived from *in vitro* transcription of the pSVA-EV71-GFP plasmid using Lipofectamine 2000. Cells were treated with TET or CEP at the indicated concentrations for 6 h, and fluorescence images were captured using a Leica DMi6000 B fluorescence microscope. Scale bar represents 100 µM. EMT was used as a control for post-entry targeting. (**D**) For each condition, the percentage of GFP (+) cells was calculated as the ratio of the number of GFP (+) cells to those stained with DAPI, compared to untreated controls (DMSO). Quantitative results are presented as mean ± SD (*n* = 4). Unpaired one-way ANOVA analysis was conducted. ****P < 0.0001; ns indicates non-significant.

To determine the specific steps of EV71 viral entry that are affected by TET and CEP, we investigated their impact on virus binding and internalization. For the virus binding assay, we incubated the cells with the virus at 4°C (to prevent virus internalization) for 1 h, either with or without the test compounds (Fig. 3A). Following this, we conducted the virus internalization assay (Fig. 3B) by increasing the temperature to 37°C for 1 h, allowing the internalization of the bound virus particles. After this step, we performed trypsinization to remove any uninternalized surface virus particles. We measured the viral RNA levels for each condition using RT-qPCR. Our results showed no detectable difference between the compound-treated group and the control group without compounds, indicating that neither TET nor CEP affected virus binding or internalization (Fig. 3A and B).

Next, a sub-genomic replicon of EV71 that encodes GFP reporter (pSVA-EV71-GFP) was used to evaluate the antiviral effects of TET and CEP in HeLa cells. This replicon system allows us to bypass receptor-mediated entry by delivering viral RNA directly into the cytoplasm through transfection (37). At 6 h post-transfection, we observed strong GFP expression in control cells that received no compounds. In contrast, emetine (EMT), a known inhibitor of EV IRES-mediated translation (38), significantly reduced the fluorescence signal. However, TET and CEP, at all tested concentrations, did not impact GFP expression (Fig. 3C and D). This suggests that these compounds do not interfere with viral translation or genome replication. Overall, our results indicate that TET and CEP do not disrupt the stages of viral attachment, internalization, or post-entry events of EV71.

### TET and CEP block the trafficking of EV71 in the endolysosomal pathway

**Fig 4:**
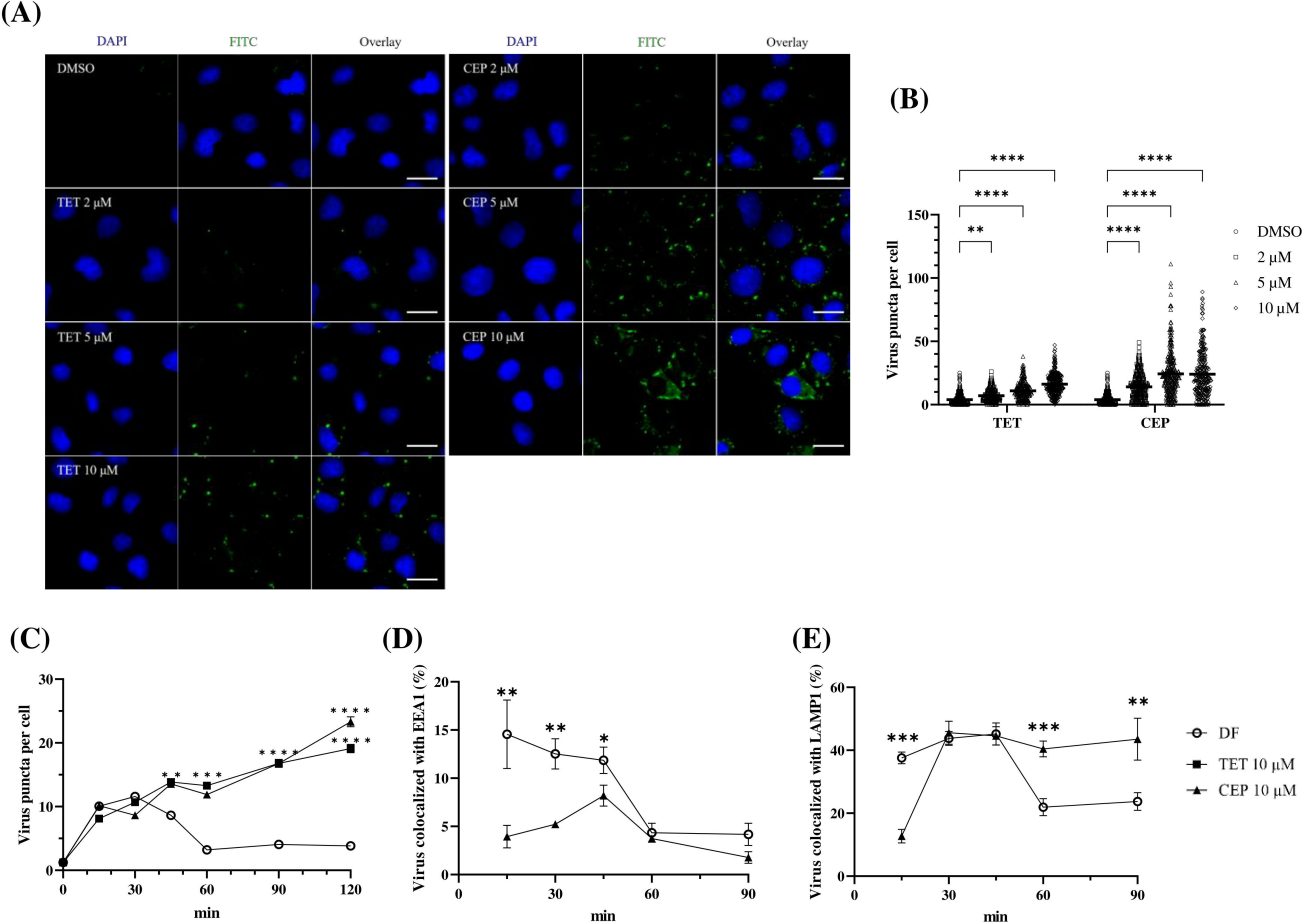
BBAs delayed EV71 entry through the endosomal compartment. (**A**) HeLa cells were inoculated with EV71 stock at an MOI of 300 at 4°C for 1 h for adsorption. Afterward, the cells were treated with TET or CEP at 10 µM for 90 min before being fixed and permeabilized. Puncta signals (green) representing EV71 virions were detected using a rabbit anti-VP2 Ab, followed by detection with a secondary Ab conjugated to Alexa Fluor 488. DAPI was used to stain the nucleus (blue). Scale bar represents 20 µM. (**B**) The number of puncta surrounding each cell was calculated based on images from five fields. (**C**) Fluorescence signals were detected at the indicated time points after adsorption with EV71. Cells were co-stained with Ab against viral VP2 and (**D**) EEA1 (indicating early endosomes) or (**E**) LAMP1 (indicating lysosomes). The portion of VP2 colocalized with EEA1 or LAMP1 is shown as the average ± SEM for more than 200 cells. ****P < 0.0001, ***P < 0.001, **P < 0.01, *P < 0.05.

Given that viral binding and internalization are independent of the antiviral effects of TET and CEP on viral entry, we next examined viral trafficking, a specific sub-step of viral entry. HeLa cells were incubated with EV71 at 4°C for 1 h, followed by treatment with either TET or CEP at concentrations of 2, 5, or 10 µM at 37°C for 90 min. The viral signal increased with higher compound concentrations, as shown by imaging analyses (Fig. 4A) and their quantification (Fig. 4B) in the IFA. Additionally, changes in viral signals were observed at various time points within a 120 min timeframe following treatment with 10 µM of TET or CEP. In the control group, viral signals peaked at 30 min, then gradually declined until reaching low levels at 60 min, where they remained thereafter. In contrast, the viral signal in the group treated with either TET or CEP increased over time (Fig. 4C; Fig. S2), suggesting that the virus may be sequestered within the endocytic organelle.

We then conducted an analysis of the motility of EV71 within the endolysosome during the viral entry process upon the treatment with CEP or left untreated (DMSO control). Using confocal microscopy, we examined the localization of viral protein in relation to two key proteins: EEA1, which is associated with early endosomes, and LAMP1, which is found in lysosomes. In the control group, we observed that the viral protein primarily colocalized with EEA1 at the 15 min mark, reaching nearly 15%, before gradually declining. In contrast, the CEP-treated group displayed significantly lower colocalization of the viral protein with EEA1 at the same time point, peaking at only about 8% at 45 min, and then decreasing over time. This observation suggests that CEP delays the trafficking of EV71 particles within the endosomes (Fig. 4D; Fig. S3). Regarding LAMP1, the viral protein colocalized with it from 30 to 45 min, reaching a peak of nearly 45% at 45 min before dropping to about 20% at later time points in the control group. In contrast, the CEP-treated group maintained a consistently high level of viral protein-LAMP1 colocalization, remaining around 40% from 30 to 90 min. This indicates that while many viral particles in the control group exited the lysosome by 60 min, those in the CEP-treated group remained mostly trapped inside the lysosomes (Fig. 4E; Fig. S3). Overall, these results imply that BBAs, specifically TET and CEP, may inhibit the movement of EV71 particles within the endolysosomal system.

### BBAs prevent the entry of EV71 by increasing lysosomal pH

**Fig 5:**
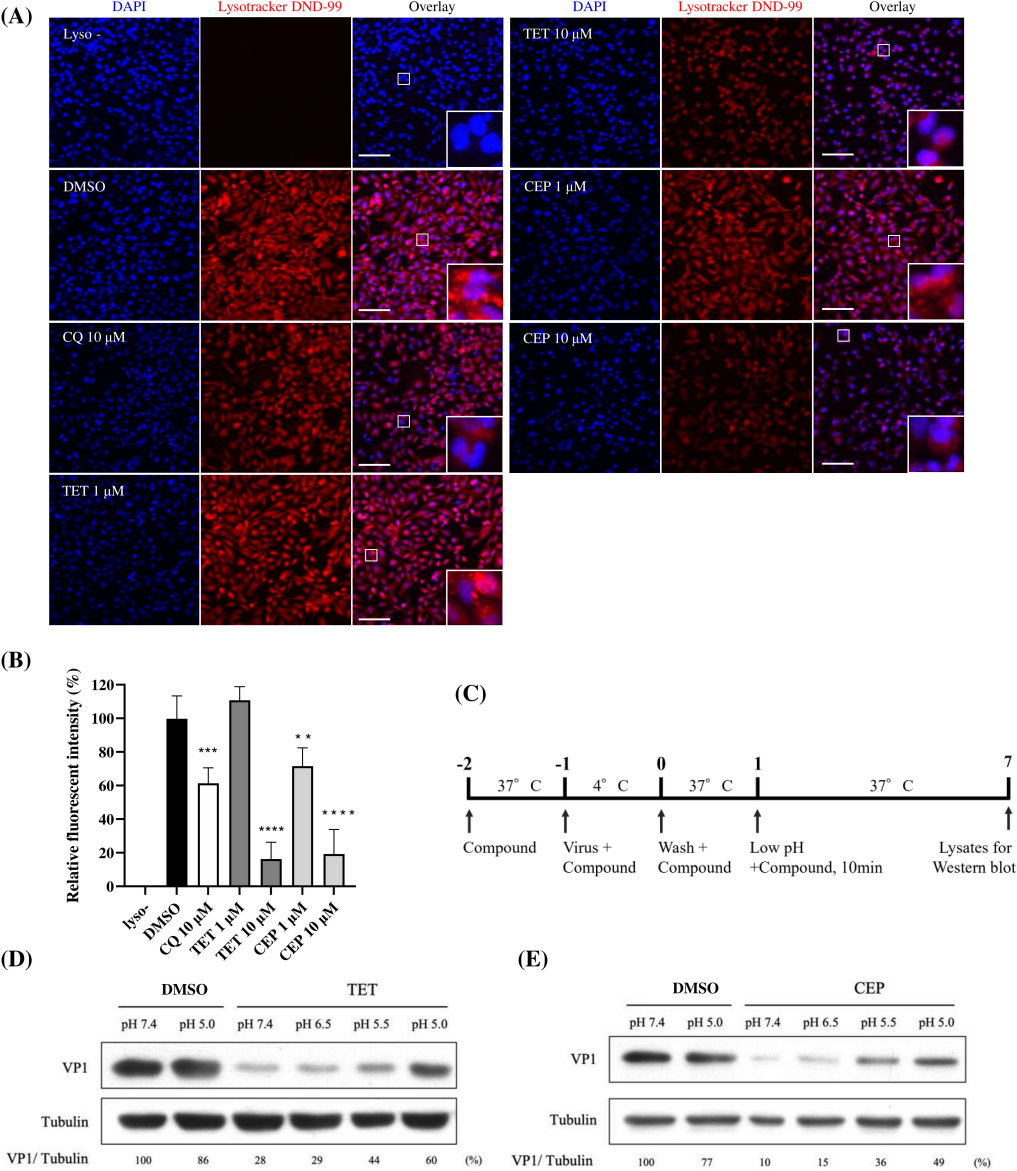
The antiviral activity of BBAs is associated with their alkalizing effect in lysosomes. (**A**) Cells were treated with CQ, TET, and CEP at the indicated concentrations for 2 h, followed by staining with 500 nM LysoTracker for 1 h. After fixation, the cells were observed using a Leica DMi6000 B fluorescence microscope. Scale bar represents 100 µM. The inset shows an enlarged field. (**B**) Fluorescence images were captured, and the intensities were quantified using ImageJ software. The fluorescence intensity from LysoTracker-stained, DMSO-treated cells was set to 100%. Results are expressed as mean ± SD (*n* = 4), with unpaired one-way ANOVA used for analysis. Significance levels are indicated as follows: ****P < 0.0001, ***P < 0.001, **P < 0.01, *P < 0.05. (**C**) Schematic timeline of drug administration and low-pH medium exposure: HeLa cells were pretreated with 10 µM TET (**D**) and 8 µM CEP (**E**) for 1 h. EV71 was used to inoculate the cells at an MOI of 0.5 in media containing the corresponding compounds at 4°C for 1 h. The cells were then washed and replaced with media containing the compounds, followed by incubation at 37°C for 1 h. Subsequently, cells were replaced with media at different pH values (7.4, 6.5, 5.5, and 5.0) containing the drugs at 37°C for 10 min, after which they were again replaced with compound-containing media (pH 7.4) for 6 h. Cell lysates were prepared for Western blot analysis to probe for viral VP1 and tubulin. The percentages shown below each lane represent the intensity of VP1 relative to that of tubulin, compared to DMSO-treated controls.

In the context of EV infections, the acidic pH of lysosomes plays crucial roles in the virus trafficking and the structural changes of virions that lead to virus uncoating (11). Since BBAs, particularly TET, CEP, and FAN, have been shown to disrupt lysosomal acidification in certain cell types (39–41), we examined whether treatment with TET or CEP caused elevation of pH in acidic cellular compartments in HeLa cells. These compartments were labeled with the LysoTracker dye, which emits red fluorescence only in acidic environments, such as endolysosomes. Chloroquine (CQ), a well-known inhibitor of endosomal acidification, was used as a control. The results indicated that, compared to the untreated control, significant decreases in fluorescence intensity were observed following treatment with TET or CEP at a concentration of 10 µM, similar to the results seen with CQ (Fig. 5A and B). Notably, treatment with CEP at 1 µM resulted in significant fluorescence reduction, while treatment with TET at the same concentration showed no detectable fluorescence reduction, which aligns with their respective antiviral activities. As TET and CEP elevated the pH levels in acidic intracellular compartments, we hypothesized that the antiviral effects of both compounds could stem from their ability to block endosomal acidification. To test this, HeLa cells were exposed to low-pH media after virus binding. The cells were pretreated with either compound before being inoculated with EV71 stock while kept at 4°C. After 1 h p.i., the inoculum was removed, and any unbound viruses were washed away. We then allowed the viruses to enter the cells by incubating them with either compound at 37°C for 1 h. Following that, the cells were incubated for 10 min in compound-containing media of pH 7.4, 6.5, 5.5, or 5.0, followed by a 6 h recovery in pH 7.4 medium that also contained the compound. We next prepared the cell lysates for Western blot analysis to assess the levels of viral protein at 6 h p.i. (Fig. 5D and E). Our findings revealed that viral replication, indicated by the levels of viral protein, could be significantly enhanced by low-pH shocks (pH 5.5 and 5.0) in a pH-dependent manner. In stark contrast, exposure to media at pH 6.5 did not lead to a significant recovery of viral replication, supporting the idea that early endosomes (with a pH of 6.0–6.5) play a minor role in viral uncoating. This result is consistent with our earlier observation that the BBA treatment caused the alkalization of the cellular acidic compartment (Fig. 5A and B). Taken together, these results suggest that TET and CEP likely target a low pH-dependent step in the viral entry process.

### Antiviral activity of TET and CEP against other EV serotypes

**Fig 6:**
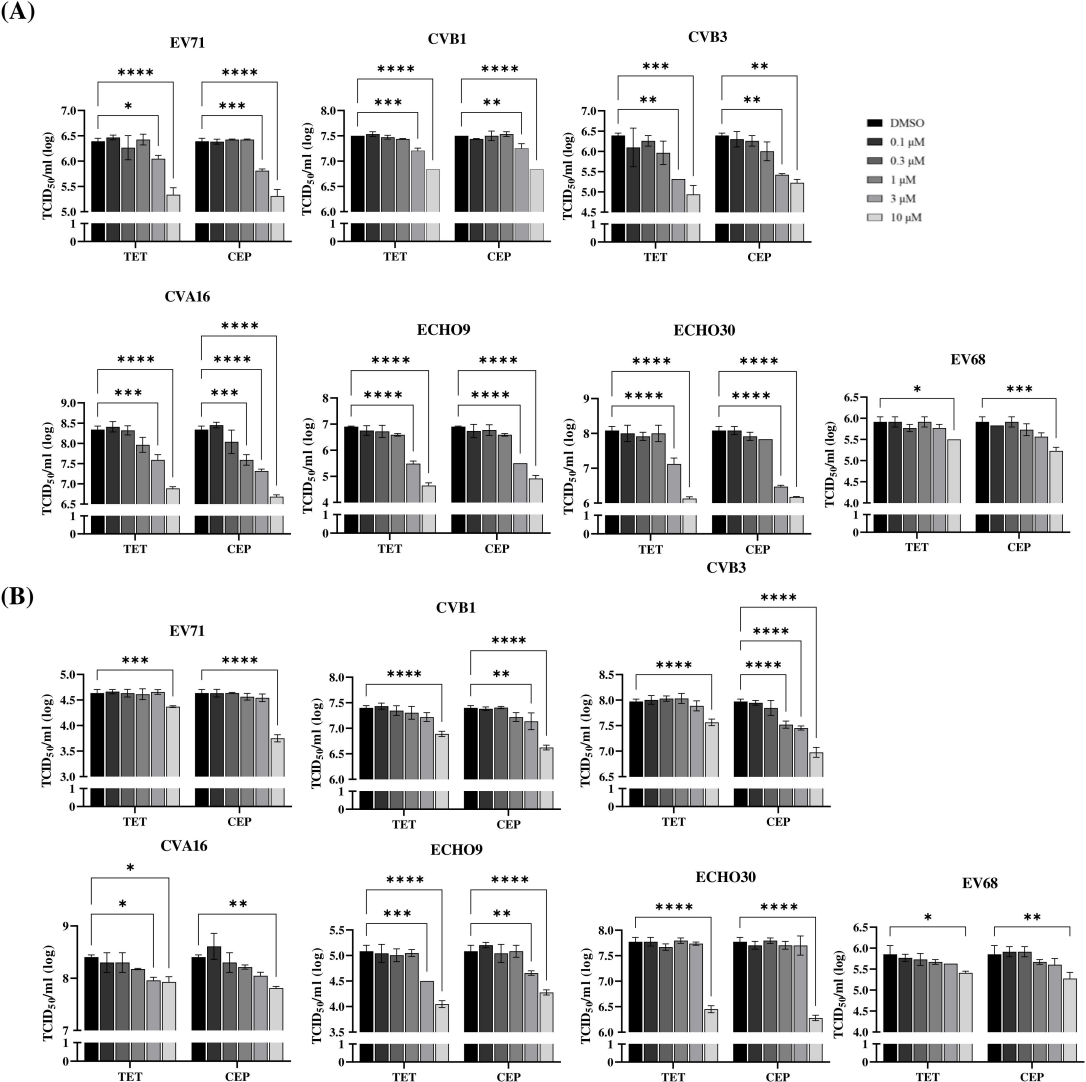
Antiviral activity of TET and CEP against a representative of EV serotypes. RD cells (**A**) and HeLa cells (**B**) were pretreated with concentrations of TET or CEP at 0.1, 0.3, 1, 3, and 10 µM for 1 h. After the pretreatment, the cells were infected with various EVs, including EV71, CVB1, CVB3, CVA16, Echo 9, Echo 30, and EV68, at an MOI of 0.1 for 1 h. The media were then replaced with fresh media containing the respective drugs, and the cells were incubated for an additional 16 h. Total viral titers were quantified using the TCID_50_ assay. The graphs present mean values along with standard deviations (*n* = 3). The TCID_50_ data were analyzed using ANOVA, with significance indicated as ****P < 0.0001, ***P < 0.001, **P < 0.01, *P < 0.05.

We investigated the antiviral effects of TET and CEP against a range of EV serotypes, including CVA16, CVB1, CVB3, Echo9, Echo30, and EV68, in addition to EV71. Total viral titers were determined using cell lysates and culture supernatants collected at 16 h p.i. Both TET and CEP demonstrated antiviral activity in HeLa and RD cells, though with varying degrees of effectiveness (Fig. 6; Table 2).

**TABLE 2:** TCID_50_-based IC_50_ values of TET and CEP against a representative panel of EVs

EV serotype	Cell line	IC_50_ (μM)^*a*^	
		TET	CEP
EV71	RD	3.2 ± 2.4	3.1 ± 2.0
	HeLa	10.1 ± 1.6	5.3 ± 1.6
CVB1	RD	3.6 ± 1.0	4.4 ± 2.1
	HeLa	5.3 ± 3.6	3.1 ± 1.4
CVB3	RD	0.6 ± 0.9	0.7 ± 0.6
	HeLa	8.0 ± 4.7	0.9 ± 0.4
CVA16	RD	1.0 ± 0.7	0.3 ± 0.2
	HeLa	0.4 ± 0.3	0.5 ± 1.0
ECHO 9	RD	0.6 ± 0.6	0.7 ± 0.8
	HeLa	2.2 ± 1.0	2.9 ± 2.7
ECHO 30	RD	1.8 ± 2.2	0.8 ± 0.5
	HeLa	5.4 ± 0.3	5.0 ± 1.8
EV68	RD	3.5 ± 2.7	2.3 ± 1.4
	HeLa	5.7 ± 1.4	1.6 ± 0.8

^*a*^ 50% inhibitory concentration.

### CEP prevents MP4 infection in mice

**Fig 7:**
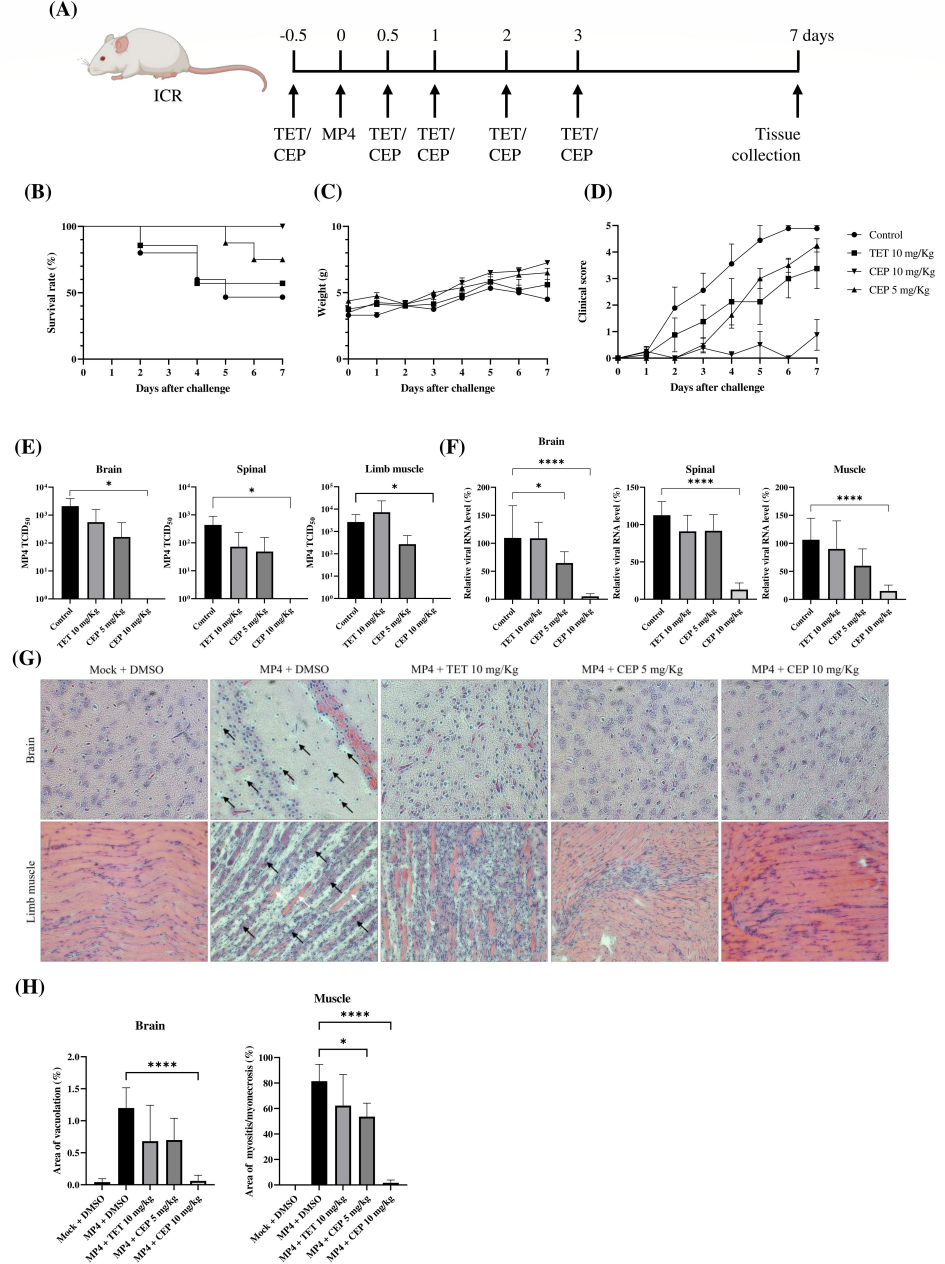
CEP treatment improved survival rate and reduced viral replication in a mouse infection model. (**A**) This schematic illustrates the treatment protocol for BBAs followed by MP4 infection in mice. One day prior to the viral challenge, 7-day-old ICR mice received intraperitoneal injections of DMSO (control), TET (10 mg/kg), or CEP (5 or 10 mg/kg), with sample sizes of *n* = 15, 8, 8, or 8, respectively. The mice were then challenged intraperitoneally with MP4 at a dose of 1 × 10^7^ TCID_50_ per mouse. At 12, 24, 48, and 72 h p.i., the mice were administered the corresponding compound and monitored for 7 days. The survival rate (**B**), weight (**C**), and clinical scores (**D**) are presented with mean values and the SEM. Survival rates were assessed using the log-rank test, with significance indicated as *P < 0.05. Following this period, the mice were euthanized, and tissues were collected for H&E staining and viral titer analysis. At 7 days p.i., 5 mice in each group were euthanized and their brain, spinal cord, and hind-limb muscle tissues were harvested. Viral titers from harvested tissues were determined by TCID₅₀ assay (**E**) and RT-qPCR (**F**). A histopathological examination of the harvested tissues was performed (**G**). In the infected group devoid of compound treatment, brain areas with vacuoles, typically filled with monocyte/macrophage infiltration, are indicated by the dark arrows. In the limb muscle of the infected control group without the compounds, the dark arrows highlight regions of myositis and myonecrosis, while the white arrow points to cells that did not undergo complete myositis and myonecrosis. Virus-infected groups administered with TET (10 mg/kg) or CEP (5 or 10 mg/kg) are indicated, along with an age-matched group treated with DMSO that is mock-infected. (**H**) The quantification of lesions in the brains and muscles was performed using ImageJ software on the groups indicated. The results are displayed as the percentage of area measured from a minimum of six locations in each of five individual mice. (**E, F, H**) Statistical comparisons were made using a one-tailed *t* test with ****P < 0.0001, ***P < 0.001, *P < 0.05.

To evaluate the *in vivo* antiviral efficacy of TET and CEP against EV71 infection, we utilized a 7-day-old ICR mouse model infected with a mouse-adapted strain MP4 of EV71 (Fig. 7A). Mice were given a single intraperitoneal dose of either TET, CEP, or vehicle control 12 h before viral inoculation. This was followed by additional doses of the same compound or a vehicle control at 12, 24, 48, and 72 h p.i. In comparison to the vehicle control group, which had a survival rate of 47% (7 out of 15 mice), the group treated with CEP at a dose of 10 mg/kg achieved an impressive 100% survival rate (8 out of 8 mice), significantly improving the survival rate (Fig. 7B). In contrast, treatment with TET at 10 mg/kg resulted in a survival rate of only 62.5% (5 out of 8 mice), while treatment with CEP at 5 mg/kg led to a survival rate of 75% (6 out of 8 mice); these results did not significantly enhance the survival rate (Fig. 7B). Additionally, there were no significant differences in body weight between the treated and control groups (Fig. 7C). Limb paralysis was observed in the control group (8 mice), as well as in the groups treated with CEP at 5 mg/kg (2 mice) and TET (3 mice), 5 days after infection. In contrast, the group treated with CEP at 10 mg/kg did not show any signs of paralysis and had significantly lower clinical scores (Fig. 7D). Viral titers and RNA levels were measured in the brain, spinal cord, and limb muscles using the TCID_50_ assay and RT-qPCR, respectively. The results showed that treatment with CEP at a dose of 10 mg/kg significantly reduced both viral titers and RNA levels compared to those of the control group, while the other treatment groups did not show this effect (Fig. 7E and F). We also performed H&E staining and analysis of brain and limb muscle tissues of the mice (Fig. 7G). In comparison to the control group devoid of compound treatment, the brain tissues of the mice treated with 10 mg/kg of CEP exhibited much milder signs of encephalitis, including reduced neutrophilic infiltration and vacuolation. Additionally, the limb muscle tissue from this treated group showed a significantly lower number of pathological features. Specifically, there was less necrosis of muscle fibers, fewer infiltrating inflammatory cells, and a reduction in fibrotic scars that typically replace damaged muscle fibers. In contrast, the tissues from the virus-infected mice that were treated with either 10 mg/kg of TET or 5 mg/kg of CEP demonstrated much less pronounced reversal of the pathological effects. In line with the findings, ImageJ quantification revealed a dose-dependent decrease in the percentage of lesion area in the hindlimb muscle tissues of mice treated with CEP at doses of 5 mg/kg and 10 mg/kg, compared to the virus-infected control group that received no treatment. Notably, a reduction in brain lesions was observed only in the mice treated with CEP at the higher dose of 10 mg/kg. In contrast, mice treated with TET at a dose of 10 mg/kg did not show a significant reduction in lesions in either the brain or hindlimb muscle (Fig. 7H). In conclusion, treatment with CEP at 10 mg/kg effectively inhibits EV71 replication and its associated pathogenesis in this murine model.

## DISCUSSION

Enteroviruses of various serotypes continue to pose significant global health threats, especially among immunocompromised adults and pediatric populations. Among these, EV71 is particularly known for causing severe neurological complications in infants and young children across the Asia-Pacific region. There are currently no approved antiviral drugs available to treat EV infections. Additionally, there is an urgent need to develop broad-spectrum anti-EV drugs, as no single EV serotype is uniquely associated with specific clinical manifestations. In this study, we explored the antiviral potential of BBAs against EVs. All tested BBAs effectively inhibited EV71 infection in a dose-dependent manner. Notably, TET and CEP showed lower IC_50_ values and higher SI values (Table 1). As a result, we have focused on TET and CEP to investigate their mechanism of action. Our findings demonstrated that TET and CEP primarily exerted their antiviral effects at the entry stage of the viral lifecycle (Fig. 4A), while excluding virus binding, internalization, and post-entry processes (Fig. 3). Specifically, both compounds immobilized viral particles during endolysosomal transport, thereby hindering effective virus uncoating (Fig. 4). This effect is at least partly attributed to the compounds’ ability to neutralize the low pH levels in endolysosomal vesicles (Fig. 5A), as acidic replenishment of the medium restored the viral infection in cells pretreated with TET or CEP (Fig. 5B). Together, these data indicate a mechanism by which BBAs neutralize the pH levels of the endolysosomal pathway and disrupt its function for transporting viruses, which is crucial for the entry of EVs. The IC_50_ values obtained from the antiviral treatment over an 8 h period (Fig. 1) are significantly lower than those measured after a 16 h treatment (Fig. 6). To further investigate this issue, we conducted an additional experiment in which the compound was replenished at 8 h p.i. to maintain a total infection period of 16 h. However, this replenishment did not restore the antiviral effect (Fig. S5; Table S2), indicating that the time-dependent reduction in antiviral activity is not related to the stability of the compound. This decrease in antiviral efficacy over time has been documented, particularly with agents targeting the early stages of replication (42, 43). Once again, the potential for compound instability has been ruled out (42), and the underlying mechanisms behind this phenomenon remain to be explored.

CEP is known to demonstrate multiple antiviral mechanisms against various enveloped viruses. It has been shown to suppress SARS-CoV-2, dengue virus, and ASFV in the early stages of infection. In contrast, CEP interferes with the virus, HSV-1, HBV, and ASFV during the mid-stage of infection. For SARS-CoV-2, CEP blocks the binding of the viral spike protein to the angiotensin-converting enzyme 2 (ACE2) receptor (21) and prevents Ca^2+^-mediated membrane fusion (22), thereby hindering viral entry. In the case of ASFV, CEP inhibits viral internalization by impairing the functions of late endosomes and lysosomes (20). Additionally, CEP is likely to inhibit dengue virus replication by interfering with the viral internalization process although the precise mechanisms remain largely unknown. Moreover, CEP impairs mid-stage viral replication through its interactions with host proteins or pathways essential for viral reproduction. In HSV-1 infections, CEP downregulates the PI3K/Akt and p38 MAPK signaling pathways, causing infected cells to halt in the G2/M phase and undergo apoptosis (24). For HBV, CEP disrupts the function of the host heat shock cognate 70 protein, which is critical for viral replication (25). While studies on HSV-1 and HBV have shown post-entry inhibition by CEP, they did not conduct time-of-addition assays, leaving open the possibility that CEP may also affect the entry stage of these infections. Similarly, CEP impairs the Hsp90-Cdc37 complex and inhibits AKT signaling, resulting in the blocking of ASFV replication during the mid-stage (44). Collectively, CEP demonstrates a range of inhibitory effects against various enveloped viruses during both the entry and post-entry stages of infection. However, most of the reports referenced are primarily based on cell culture studies, except for the ones involving SARS-CoV-2, where *in vivo* infections and treatments were conducted to observe the effects in mice (45). Further investigation is required to validate the *in vivo* antiviral effects of CEP across different virus infection models.

Among the BBAs, it was reported that FAN has inhibitory effects against EVs of various serotypes by targeting the early stages of infection (30). This is the only report indicating that a BBA possesses antiviral activity against non-enveloped viruses, specifically EVs. The IC_50_ values of FAN against different EVs align with our findings. When selecting FAN-resistant EV71, mutations were observed in structural proteins, with the E145G mutant in VP1 displaying particularly strong resistance. The variation of E145G can bind the virion to the heparan sulfate (HS) attachment receptor, which enhances the virus’s infectivity (46, 47). Moreover, our structure-based docking study shows that FAN binds effectively to VP1 E145, but it cannot bind to VP1 E145G. On the other hand, CEP does not bind to either VP1 E145 or VP1 E145G (Fig. S6). This indicates that the VP1 protein could be a potential target for FAN, distinguishing it from our findings that CEP action is independent of viral binding (Fig. 3A).

BBAs exhibit structural and physicochemical properties characteristic of lysomotropic cationic amphiphilic drugs (CADs) as shown in Fig. 1A (39, 48, 49). CADs consist of a hydrophobic aromatic ring along with hydrophilic segments that contain an ionizable amine functional group. Due to their amphiphilic nature, CADs can easily permeate cellular membranes, undergo protonation, and become trapped within acidic intracellular compartments, such as late endosomes and lysosomes. This accumulation can cause alkalization (50) and impair the endolysosomal pathway (51), which is essential for EV entry. Two quantitative measurements of the physicochemical properties of CADs are the calculated logP (ClogP) value, which indicates the compounds’ lipophilicity, and the basic pKa, the acid dissociation constant for the conjugate acid of the weak base. These measurements influence both the extent of lysosomal trapping and the kinetics of passive permeation. Research has indicated that compounds with a ClogP greater than 3 and a basic pKa greater than 7.4 are often classified as CADs (51). However, some studies suggest that less stringent criteria—a ClogP greater than 3 and a basic pKa greater than 6—can also be applicable. Both TET, which has a ClogP of 3.23 and a basic pKa of 7.70, and CEP, with a ClogP of 6.29 and a basic pKa of 7.61, meet these criteria (Table S1) (52). This may explain a mechanism involved in the pH elevation of the endolysosomal system by TET or CEP.

Two-pore channels (TPCs) and transient receptor potential mucolipin (TRPML) are important ion channels located within the endolysosomal system. They primarily regulate calcium flux, which, in turn, influences endolysosomal pH levels and vesicular dynamics. TPCs and TRPML are essential for the entry and intracellular trafficking of various pathogens including viruses (53, 54). Previous reports have identified TET as a TPC inhibitor that can hinder the entry of the Ebola virus and coronaviruses (18, 55, 56). Molecular docking analyses suggest that CEP may also bind TPC2, thereby impairing its functions (57), although this has not yet been empirically validated. On the other hand, BER is known to block the infection of SARS-CoV-2 and flaviviruses by disrupting the endolysosomal trafficking of viral receptors mediated by TPRMLs (57). However, BER exhibited relatively modest antiviral effects in the present study (Fig. 1B through D). We proposed that the primary inhibitory mechanism against EV71 might also involve TPCs though it’s less likely to involve TRPMLs. Further mechanistic investigations are needed to determine if CEP and TET influence the acidity-dependent endocytosis of EV entry via TPCs.

We demonstrated that the prophylactic administration of CEP in a mouse model provided protection against a lethal EV71 challenge by decreasing mortality, viral load, and tissue damage (Fig. 7). To the best of our knowledge, this is the first study to identify a BBA as an effective inhibitor of EV71 *in vivo*. CEP is approved in Japan for the treatment of radioactive leukopenia, alopecia, venomous snake bites, and chronic exudative otitis media (58–60). Although the exact mechanisms of action of CEP are not fully clarified, repurposing an approved drug significantly accelerates the process of advancing a candidate drug into clinical practice. This is due to the fact that the safety and pharmacokinetic profiles of these approved drugs have already undergone extensive evaluation, paving the way for a more efficient transition. Studies have shown that intravenous administration of 100 mg of CEP in humans can achieve a maximum concentration (*C*_max_) of drug in serum of 1,464 ± 364 ng/mL, which is approximately 2.41 µM (61). In mice, an intraperitoneal injection at a dose of 21 mg/kg results in a *C*_max_ of 874 ng/mL (approximately 1.44 µM) (62). The *C*_max_ values of CEP in humans and mice are higher than the *in vitro* IC_50_ values observed in the 8 h antiviral studies (Fig. 1; Table 1). However, these *C*_max_ values are similar to or lower than those recorded in the antiviral studies conducted over a 16 h infection period (Fig. 6; Table 2). Therefore, successful clinical translation will likely depend on dosing strategies that maintain effective drug levels over time. A notable challenge with the oral delivery of CEP is its poor solubility, which significantly hinders its effectiveness *in vivo* (63). To address this issue, further research is essential to improve its bioavailability, particularly through oral administration.

In sum, our study demonstrates that BBAs, particularly CEP, displayed broad-spectrum activity against multiple EV serotypes (Fig. 6), likely by disrupting lysosomal acidification and interfering with virus trafficking, resulting in effective inhibition of viral entry. The pH-dependent endolysosomal pathway is a common mechanism shared by many different virus groups. Therefore, further investigation into CEP’s antiviral spectrum via this pathway is warranted. Additionally, as a host-directed antiviral agent, CEP presents a greater challenge for the development of drug resistance. These findings broaden the scope of BBA-based antiviral strategies and strongly support the need for further optimization and clinical investigation of CEP and similar compounds. In a landscape where therapeutic options are limited, utilizing the lysosomotropic and host-targeting properties of BBAs could provide an effective method for combating EV infections.

## SUPPLEMENTAL MATERIAL

Supplemental material aac.00764-25-s0001.pdf Tables S1 and S2; Fig. S1 to S6.

ASM does not own the copyrights to Supplemental Material that may be linked to, or accessed through, an article. The authors have granted ASM a non-exclusive, world-wide license to publish the Supplemental Material files. Please contact the corresponding author directly for reuse.

## References

[1] Ooi MH, Wong SC, Lewthwaite P, Cardosa MJ, Solomon T. 2010. Clinical features, diagnosis, and management of enterovirus 71. Lancet Neurol 9:1097–1105. doi:10.1016/S1474-4422(10)70209-X 20965438

[2] Solomon T, Lewthwaite P, Perera D, Cardosa MJ, McMinn P, Ooi MH. 2010. Virology, epidemiology, pathogenesis, and control of enterovirus 71. Lancet Infect Dis 10:778–790. doi:10.1016/S1473-3099(10)70194-8 20961813

[3] Liu D-P, Wang T-A, Huang W-T, Chang L-Y, Wang E-T, Cheng S-H, Yang M-C. 2016. Disease burden of enterovirus infection in Taiwan: implications for vaccination policy. Vaccine (Auckl) 34:974–980. doi:10.1016/j.vaccine.2015.12.026 26768128

[4] Zhu P, Ji W, Li D, Li Z, Chen Y, Dai B, Han S, Chen S, Jin Y, Duan G. 2023. Current status of hand-foot-and-mouth disease. J Biomed Sci 30:15. doi:10.1186/s12929-023-00908-4 36829162 PMC9951172

[5] Fan S, Liao Y, Jiang G, Jiang L, Wang L, Xu X, Feng M, Yang E, Zhang Y, Cui W, Li Q. 2020. Study of integrated protective immunity induced in rhesus macaques by the intradermal administration of a bivalent EV71-CA16 inactivated vaccine. Vaccine (Auckl) 38:2034–2044. doi:10.1016/j.vaccine.2019.12.057 31982260

[6] Yamayoshi S, Iizuka S, Yamashita T, Minagawa H, Mizuta K, Okamoto M, Nishimura H, Sanjoh K, Katsushima N, Itagaki T, Nagai Y, Fujii K, Koike S. 2012. Human SCARB2-dependent infection by coxsackievirus A7, A14, and A16 and enterovirus 71. J Virol 86:5686–5696. doi:10.1128/JVI.00020-12 22438546 PMC3347270

[7] Lin Y-W, Lin H-Y, Tsou Y-L, Chitra E, Hsiao K-N, Shao H-Y, Liu C-C, Sia C, Chong P, Chow Y-H. 2012. Human SCARB2-mediated entry and endocytosis of EV71. PLoS One 7:e30507. doi:10.1371/journal.pone.0030507 22272359 PMC3260287

[8] Kobayashi K, Koike S. 2020. Cellular receptors for enterovirus A71. J Biomed Sci 27:23. doi:10.1186/s12929-020-0615-9 31924205 PMC6954530

[9] Galitska G, Jassey A, Wagner MA, Pollack N, Miller K, Jackson WT. 2023. Enterovirus D68 capsid formation and stability requires acidic compartments. mBio 14:e0214123. doi:10.1128/mbio.02141-23 37819109 PMC10653823

[10] Liu Y, Sheng J, van Vliet ALW, Buda G, van Kuppeveld FJM, Rossmann MG. 2018. Molecular basis for the acid-initiated uncoating of human enterovirus D68. Proc Natl Acad Sci USA 115:E12209–E12217. doi:10.1073/pnas.1803347115 30530701 PMC6310856

[11] Baggen J, Thibaut HJ, Strating J, van Kuppeveld FJM. 2018. The life cycle of non-polio enteroviruses and how to target it. Nat Rev Microbiol 16:368–381. doi:10.1038/s41579-018-0005-4 29626210

[12] van der Linden L, Wolthers KC, van Kuppeveld FJM. 2015. Replication and inhibitors of enteroviruses and parechoviruses. Viruses 7:4529–4562. doi:10.3390/v7082832 26266417 PMC4576193

[13] Paudel KR, Karki R, Kim DW. 2016. Cepharanthine inhibits in vitro VSMC proliferation and migration and vascular inflammatory responses mediated by RAW264.7. Toxicol In Vitro 34:16–25. doi:10.1016/j.tiv.2016.03.010 27021874

[14] Gülçin I, Elias R, Gepdiremen A, Chea A, Topal F. 2010. Antioxidant activity of bisbenzylisoquinoline alkaloids from Stephania rotunda: cepharanthine and fangchinoline. J Enzyme Inhib Med Chem 25:44–53. doi:10.3109/14756360902932792 20030508

[15] Harada T, Harada K, Ueyama Y. 2012. The enhancement of tumor radioresponse by combined treatment with cepharanthine is accompanied by the inhibition of DNA damage repair and the induction of apoptosis in oral squamous cell carcinoma. Int J Oncol 41:565–572. doi:10.3892/ijo.2012.1501 22664937

[16] Samita F, Ochieng CO, Owuor PO, Manguro LOA, Midiwo JO. 2017. Isolation of a new β-carboline alkaloid from aerial parts of Triclisia sacleuxii and its antibacterial and cytotoxicity effects. Nat Prod Res 31:529–536. doi:10.1080/14786419.2016.1201666 27373319

[17] Desgrouas C, Chapus C, Desplans J, Travaille C, Pascual A, Baghdikian B, Ollivier E, Parzy D, Taudon N. 2014. In vitro antiplasmodial activity of cepharanthine. Malar J 13:327. doi:10.1186/1475-2875-13-327 25145413 PMC4152577

[18] Sakurai Y, Kolokoltsov AA, Chen C-C, Tidwell MW, Bauta WE, Klugbauer N, Grimm C, Wahl-Schott C, Biel M, Davey RA. 2015. Ebola virus. Two-pore channels control Ebola virus host cell entry and are drug targets for disease treatment. Science 347:995–998. doi:10.1126/science.1258758 25722412 PMC4550587

[19] Liu J, Wang F, Wang X, Fan S, Li Y, Xu M, Hu H, Liu K, Zheng B, Wang L, Zhang H, Li J, Li W, Zhang W, Hu Z, Cao R, Zhuang X, Wang M, Zhong W. 2023. Antiviral effects and tissue exposure of tetrandrine against SARS-CoV-2 infection and COVID-19. MedComm (2020) 4:e206. doi:10.1002/mco2.206 36699286 PMC9851407

[20] Zhu J, Chen H, Gao F, Jian W, Huang G, Sunkang Y, Chen X, Liao M, Zhang K, Qi W, Huang L. 2024. Bis-benzylisoquinoline alkaloids inhibit African swine fever virus internalization and replication by impairing late endosomal/lysosomal function. J Virol 98:e0032724. doi:10.1128/jvi.00327-24 39082785 PMC11334529

[21] Ohashi H, Watashi K, Saso W, Shionoya K, Iwanami S, Hirokawa T, Shirai T, Kanaya S, Ito Y, Kim KS, et al.. 2021. Potential anti-COVID-19 agents, cepharanthine and nelfinavir, and their usage for combination treatment. iScience 24:102367. doi:10.1016/j.isci.2021.102367 33817567 PMC7997640

[22] He C-L, Huang L-Y, Wang K, Gu C-J, Hu J, Zhang G-J, Xu W, Xie Y-H, Tang N, Huang A-L. 2021. Identification of bis-benzylisoquinoline alkaloids as SARS-CoV-2 entry inhibitors from a library of natural products. Signal Transduct Target Ther 6:131. doi:10.1038/s41392-021-00531-5 33758167 PMC7985570

[23] Wen X, Zhang L, Liu Q, Xiao X, Huang W, Wang Y. 2022. Screening and identification of HTNV_pv_ entry inhibitors with high-throughput pseudovirus-based chemiluminescence. Virol Sin 37:531–537. doi:10.1016/j.virs.2022.04.015 35513270 PMC9437608

[24] Liu Y, Chen L, Liu W, Li D, Zeng J, Tang Q, Zhang Y, Luan F, Zeng N. 2021. Cepharanthine suppresses herpes simplex virus type 1 replication through the downregulation of the PI3K/Akt and p38 MAPK signaling pathways. Front Microbiol 12:795756. doi:10.3389/fmicb.2021.795756 34956164 PMC8696181

[25] Zhou Y-B, Wang Y-F, Zhang Y, Zheng L-Y, Yang X-A, Wang N, Jiang J-H, Ma F, Yin D-T, Sun C-Y, Wang Q-D. 2012. In vitro activity of cepharanthine hydrochloride against clinical wild-type and lamivudine-resistant hepatitis B virus isolates. Eur J Pharmacol 683:10–15. doi:10.1016/j.ejphar.2012.02.030 22387093 PMC7094493

[26] Okamoto M, Ono M, Baba M. 1998. Potent inhibition of HIV type 1 replication by an antiinflammatory alkaloid, cepharanthine, in chronically infected monocytic cells. AIDS Res Hum Retroviruses 14:1239–1245. doi:10.1089/aid.1998.14.1239 9764907

[27] Matsuda K, Hattori S, Komizu Y, Kariya R, Ueoka R, Okada S. 2014. Cepharanthine inhibited HIV-1 cell-cell transmission and cell-free infection via modification of cell membrane fluidity. Bioorg Med Chem Lett 24:2115–2117. doi:10.1016/j.bmcl.2014.03.041 24704028

[28] Zhang W, Shen H, Wang M, Fan X, Wang S, Wuri N, Zhang B, He H, Zhang C, Liu Z, Liao M, Zhang J, Li Y, Zhang J. 2023. Fangchinoline inhibits the PEDV replication in intestinal epithelial cells via autophagic flux suppression. Front Microbiol 14:1164851. doi:10.3389/fmicb.2023.1164851 37485535 PMC10360400

[29] Huang L, Li H, Ye Z, Xu Q, Fu Q, Sun W, Qi W, Yue J. 2021. Berbamine inhibits Japanese encephalitis virus (JEV) infection by compromising TPRMLs-mediated endolysosomal trafficking of low-density lipoprotein receptor (LDLR). Emerg Microbes Infect 10:1257–1271. doi:10.1080/22221751.2021.1941276 34102949 PMC8238074

[30] Zhang Q-Y, Li J-Q, Li Q, Zhang Y, Zhang Z-R, Li X-D, Zhang H-Q, Deng C-L, Yang F-X, Xu Y, Zhang B. 2024. Identification of fangchinoline as a broad-spectrum enterovirus inhibitor through reporter virus based high-content screening. Virol Sin 39:301–308. doi:10.1016/j.virs.2024.02.006 38452856 PMC11074637

[31] Tseng K-C, Hsu B-Y, Ling P, Lu W-W, Lin C-W, Kung S-H. 2022. Antidepressant sertraline is a broad-spectrum inhibitor of enteroviruses targeting viral entry through neutralization of endolysosomal acidification. Viruses 14:109. doi:10.3390/v14010109 35062313 PMC8780434

[32] Hou H-Y, Lu W-W, Wu K-Y, Lin C-W, Kung S-H. 2016. Idarubicin is a broad-spectrum enterovirus replication inhibitor that selectively targets the virus internal ribosomal entry site. J Gen Virol 97:1122–1133. doi:10.1099/jgv.0.000431 26879094

[33] Reed LJ, Muench H. 1938. A simple method of estimating fifty per cent endpoints12. Am J Epidemiol 27:493–497. doi:10.1093/oxfordjournals.aje.a118408

[34] Wang Y-F, Chou C-T, Lei H-Y, Liu C-C, Wang S-M, Yan J-J, Su I-J, Wang J-R, Yeh T-M, Chen S-H, Yu C-K. 2004. A mouse-adapted enterovirus 71 strain causes neurological disease in mice after oral infection. J Virol 78:7916–7924. doi:10.1128/JVI.78.15.7916-7924.2004 15254164 PMC446098

[35] Himmler GE, Mladinich MC, Conde JN, Gorbunova EE, Lindner MR, Kim HK, Mackow ER. 2025. Passage-attenuated Powassan virus LI9P protects mice from lethal LI9 challenge and links envelope residue D308 to neurovirulence. mBio 16:e0006525. doi:10.1128/mbio.00065-25 39998203 PMC11980571

[36] Lu M-Y, Lin Y-L, Kuo Y, Chuang C-F, Wang J-R, Liao F. 2021. Muscle tissue damage and recovery after EV71 infection correspond to dynamic macrophage phenotypes. Front Immunol 12:648184. doi:10.3389/fimmu.2021.648184 34305887 PMC8299204

[37] Gigante A, Li M, Junghänel S, Hirschhäuser C, Knauer S, Schmuck C. 2019. Non-viral transfection vectors: are hybrid materials the way forward? Medchemcomm 10:1692–1718. doi:10.1039/c9md00275h 32180915 PMC7053704

[38] Tang Q, Li S, Du L, Chen S, Gao J, Cai Y, Xu Z, Zhao Z, Lan K, Wu S. 2020. Emetine protects mice from enterovirus infection by inhibiting viral translation. Antiviral Res 173:104650. doi:10.1016/j.antiviral.2019.104650 31734270

[39] Lyu J, Yang EJ, Head SA, Ai N, Zhang B, Wu C, Li R-J, Liu Y, Yang C, Dang Y, Kwon HJ, Ge W, Liu JO, Shim JS. 2017. Pharmacological blockade of cholesterol trafficking by cepharanthine in endothelial cells suppresses angiogenesis and tumor growth. Cancer Lett 409:91–103. doi:10.1016/j.canlet.2017.09.009 28923401 PMC5634947

[40] He L-N, Liu Y-J, Jiang J-B, Wang D-Y, Li Y-L, Zeng S-J, Guo Z, Yao P-Y, Lin Z-C, Lv S-X, Liu X-Y, Guo W, Liu F, Du B-Y, Zhao T-X, Xiao J-Y, Shi Y-F, Wang K. 2025. Tetrandrine augments melanoma cell immunogenicity via dual inhibition of autophagic flux and proteasomal activity enhancing MHC-I presentation. Acta Pharmacol Sin 46:2056–2072. doi:10.1038/s41401-025-01507-9 40016522 PMC12205077

[41] Ren Z, Song Y, Xian J, Liao Y, Zhan Y, Zhao T, Wang H, Jiang J, Xu M, Jiang Y, Liu X, Wei X, Wang K, Xiao J. 2023. Identification of Fangchinoline as a novel autophagy inhibitor with an adjuvant of chemotherapy against lung cancer. Toxicol Appl Pharmacol 477:116679. doi:10.1016/j.taap.2023.116679 37689368

[42] Wolkerstorfer A, Kurz H, Bachhofner N, Szolar OHJ. 2009. Glycyrrhizin inhibits influenza A virus uptake into the cell. Antiviral Res 83:171–178. doi:10.1016/j.antiviral.2009.04.012 19416738 PMC7126985

[43] Zhang L, Zhou D, Li Q, Zhu S, Imran M, Duan H, Cao S, Ke S, Ye J. 2021. The antiviral effect of novel steroidal derivatives on flaviviruses. Front Microbiol 12:727236. doi:10.3389/fmicb.2021.727236 34690968 PMC8527100

[44] Su G, Su L, Luo D, Yang X, Liu Z, Lin Q, An T, Weng C, Chen W, Zeng Z, Chen J. 2024. Cepharanthine inhibits African swine fever virus replication by suppressing AKT-associated pathways through disrupting Hsp90-Cdc37 complex. Int J Biol Macromol 282:137070. doi:10.1016/j.ijbiomac.2024.137070 39486740

[45] Zhang S, Huang W, Ren L, Ju X, Gong M, Rao J, Sun L, Li P, Ding Q, Wang J, Zhang QC. 2022. Comparison of viral RNA-host protein interactomes across pathogenic RNA viruses informs rapid antiviral drug discovery for SARS-CoV-2. Cell Res 32:9–23. doi:10.1038/s41422-021-00581-y 34737357 PMC8566969

[46] Nishimura Y, Lee H, Hafenstein S, Kataoka C, Wakita T, Bergelson JM, Shimizu H. 2013. Enterovirus 71 binding to PSGL-1 on leukocytes: VP1-145 acts as a molecular switch to control receptor interaction. PLoS Pathog 9:e1003511. doi:10.1371/journal.ppat.1003511 23935488 PMC3723564

[47] Kobayashi K, Sudaka Y, Takashino A, Imura A, Fujii K, Koike S. 2018. Amino acid variation at VP1-145 of enterovirus 71 determines attachment receptor usage and neurovirulence in human scavenger receptor B2 transgenic mice. J Virol 92:15. doi:10.1128/JVI.00681-18 PMC6052303 29848584

[48] Shiraishi N, Akiyama S, Nakagawa M, Kobayashi M, Kuwano M. 1987. Effect of bisbenzylisoquinoline (biscoclaurine) alkaloids on multidrug resistance in KB human cancer cells. Cancer Res 47:2413–2416. 3567927

[49] Nagatsuka S, Nakazawa T. 1982. Effects of membrane-stabilizing agents, cholesterol and cepharanthin, on radiation-induced lipid peroxidation and permeability in liposomes. Biochimica Biophysica Acta 691:171–177. doi:10.1016/0005-2736(82)90226-7

[50] Salata C, Calistri A, Parolin C, Baritussio A, Palù G. 2017. Antiviral activity of cationic amphiphilic drugs. Expert Rev Anti Infect Ther 15:483–492. doi:10.1080/14787210.2017.1305888 28286997 PMC7103695

[51] Muehlbacher M, Tripal P, Roas F, Kornhuber J. 2012. Identification of drugs inducing phospholipidosis by novel in vitro data. ChemMedChem 7:1925–1934. doi:10.1002/cmdc.201200306 22945602 PMC3533795

[52] Lu S, Nixon RA. 2016. Autophagy Enhancers, are we there Yet?, p 315–356. In Lysosomes: biology, diseases, and therapeutics

[53] Marchant JS, Patel S. 2015. Two-pore channels at the intersection of endolysosomal membrane traffic. Biochem Soc Trans 43:434–441. doi:10.1042/BST20140303 26009187 PMC4730950

[54] Ruas M, Rietdorf K, Arredouani A, Davis LC, Lloyd-Evans E, Koegel H, Funnell TM, Morgan AJ, Ward JA, Watanabe K, Cheng X, Churchill GC, Zhu MX, Platt FM, Wessel GM, Parrington J, Galione A. 2010. Purified TPC isoforms form NAADP receptors with distinct roles for Ca^2+^ signaling and endolysosomal trafficking. Curr Biol 20:703–709. doi:10.1016/j.cub.2010.02.049 20346675 PMC2861162

[55] Gunaratne GS, Yang Y, Li F, Walseth TF, Marchant JS. 2018. NAADP-dependent Ca^2+^ signaling regulates Middle East respiratory syndrome-coronavirus pseudovirus translocation through the endolysosomal system. Cell Calcium 75:30–41. doi:10.1016/j.ceca.2018.08.003 30121440 PMC6251489

[56] Gunaratne GS, Brailoiu E, He S, Unterwald EM, Patel S, Slama JT, Walseth TF, Marchant JS. 2021. Essential requirement for JPT2 in NAADP-evoked Ca^2+^ signaling. Sci Signal 14:eabd5605. doi:10.1126/scisignal.abd5605 33758061 PMC8315109

[57] Hijikata A, Shionyu-Mitsuyama C, Nakae S, Shionyu M, Ota M, Kanaya S, Hirokawa T, Nakajima S, Watashi K, Shirai T. 2022. Evaluating cepharanthine analogues as natural drugs against SARS-CoV-2. FEBS Open Bio 12:285–294. doi:10.1002/2211-5463.13337 PMC8727928 34850606

[58] Kumazawa Y, Kaneko M, Inagaki K, Matsuzaki N, Nomoto K. 1990. Accelerated recovery from γ-irradiation-induced leukopenia in mice by the biscoclaurine alkaloid, cepharanthin — Comparison with recombinant human granulocyte colony-stimulating factor. Int J Immunopharmacol 12:523–530. doi:10.1016/0192-0561(90)90116-5 1698736

[59] Bailly C. 2019. Cepharanthine: an update of its mode of action, pharmacological properties and medical applications. Phytomedicine 62:152956. doi:10.1016/j.phymed.2019.152956 31132753 PMC7126782

[60] Rogosnitzky M, Okediji P, Koman I. 2020. Cepharanthine: a review of the antiviral potential of a Japanese-approved alopecia drug in COVID-19. Pharmacol Rep 72:1509–1516. doi:10.1007/s43440-020-00132-z 32700247 PMC7375448

[61] Yasuda K, Moro M, Akasu M, Ohnishi A. 1989. Pharmacokinetic disposition of cepharanthin following single and multiple intravenous doses in healthy subjects. Rinsho yakuri/Jpn J Clin Pharmacol Ther 20:741–749. doi:10.3999/jscpt.20.741

[62] Desgrouas C, Desbordes M, Dormoi J, Ollivier E, Parzy D, Taudon N. 2014. Quantitative analysis of cepharanthine in plasma based on semiautomatic microextraction by packed sorbent combined with liquid chromatography. J Anal Methods Chem 2014:695231. doi:10.1155/2014/695231 24693462 PMC3945228

[63] Liang D, Li Q, Du L, Dou G. 2022. Pharmacological effects and clinical prospects of cepharanthine. Molecules 27:8933. doi:10.3390/molecules27248933 36558061 PMC9782661

