# Supplementary material for: Cepharanthine inhibits enterovirus entry by endolysosomal deacidification and exhibits protective activity in vivo
Source: Antimicrob Agents Chemother. 2025 Oct 29;69(12):e00764-25. doi: 10.1128/aac.00764-25 (PMC12691679; doi:10.1128/aac.00764-25)
Supplement: Supplemental material — Tables S1 and S2; Fig. S1 to S6. [file aac.00764-25-s0001.pdf]

TABLE S1 Physicochemical properties of BBAs that influence lysosomal trapping and cellular distribution

Compound	ClogP	Basic pKa
TET	3.23	7.70
CEP	6.29	7.61
FAN	3.35	7.71
BER	5.78	8.17

TABLE S2 $TCID_{50}$-based IC_{50} values of TET and CEP against a representative panel of EVs (8+8 h)

EV serotype	Cell line	IC_{50} (μM)[a]	
		TET	CEP
EV71	RD	2.1 ± 2.8	1.4 ± 1.5
	HeLa	9.7 ± 5.3	9.2 ± 6.3
EV68	RD	4.9 ± 1.5	3.1 ± 2.3
	HeLa	9.3 ± 3.1	6.2 ± 1.5

[a] 50% inhibitory concentration

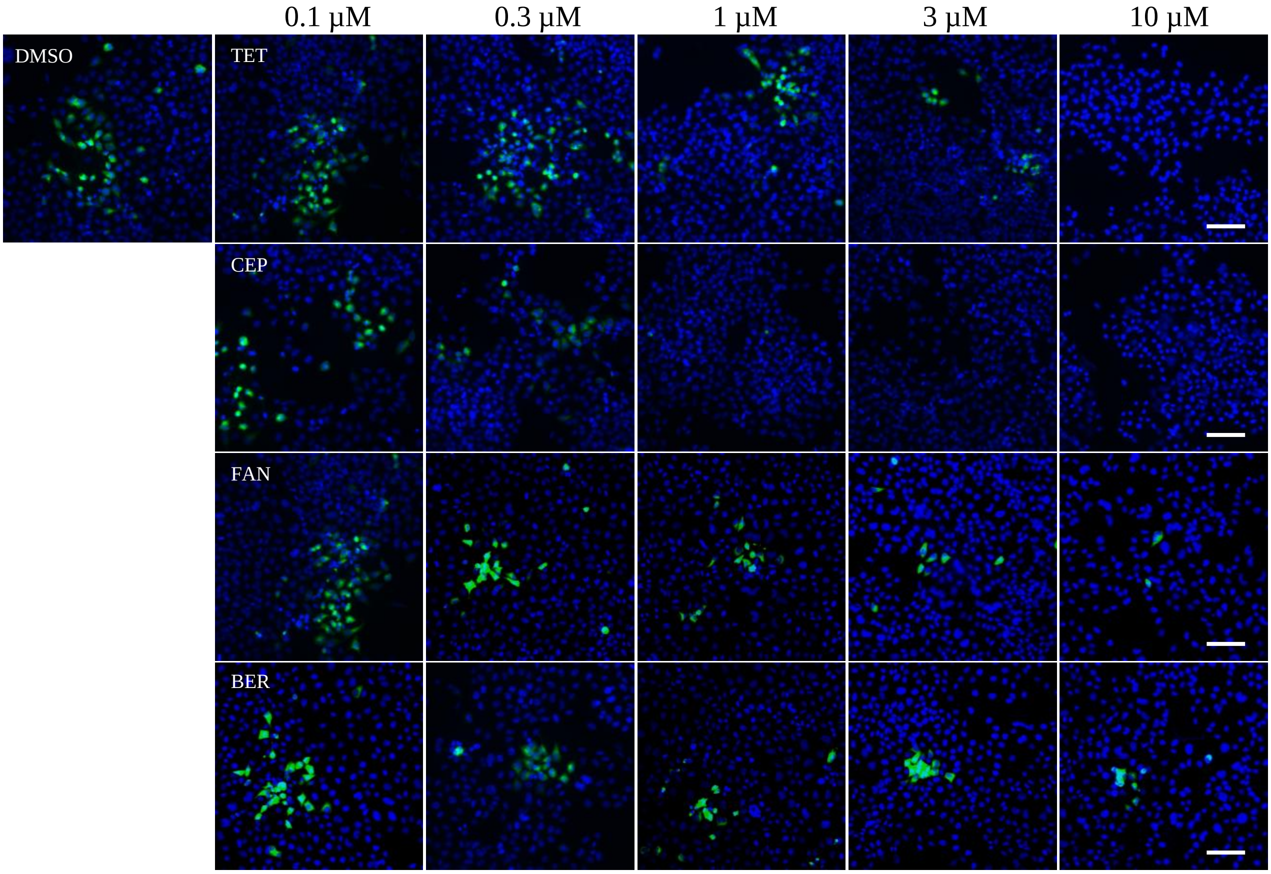

Figure S1. Representative IFA images showed dose-dependent inhibition of EV71 infection by BBAs. HeLa cells were pre-treated for 1 h with TET, CEP, FAN, or BER at concentrations indicated. Following this, the cells were infected with EV71 at an MOI of 0.1 in the presence of the same compound. At 12 h p.i., the cells were fixed, permeabilized, and stained for the viral capsid protein VP1 (green), while the nuclei were stained with DAPI (blue). For each condition, five random fields were captured using identical exposure settings on a Leica fluorescence microscope. Scale bar indicate 100 µm.

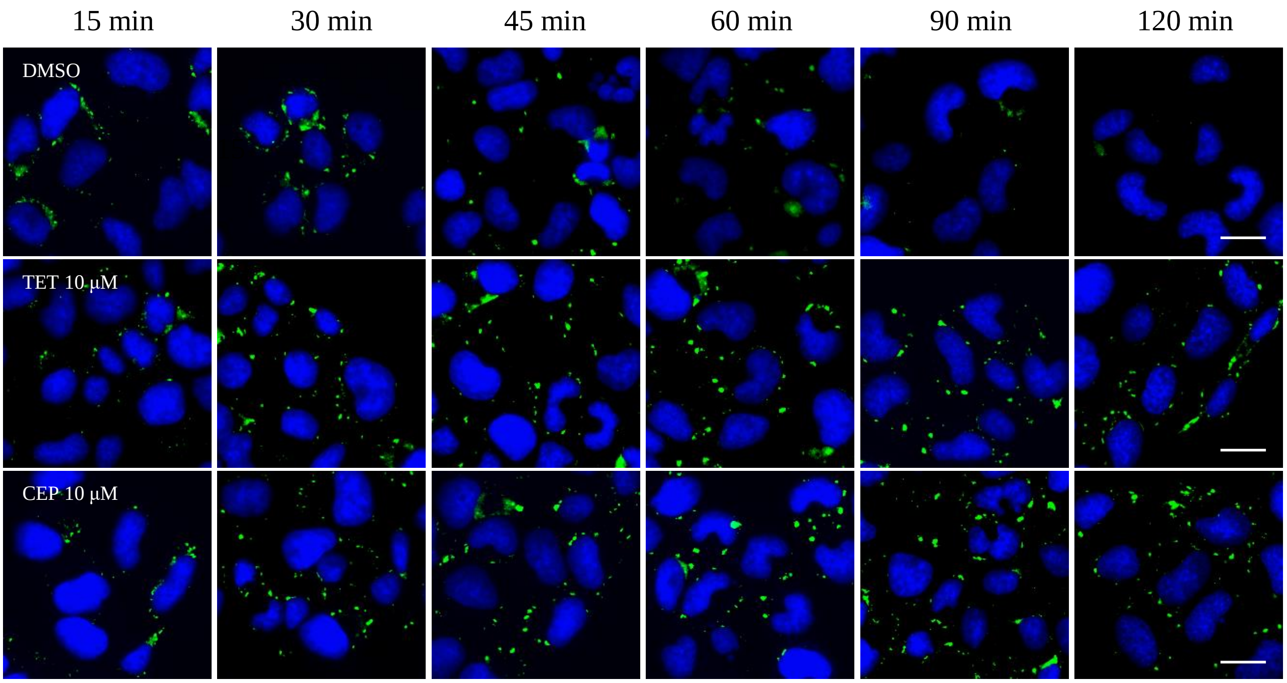

Figure S2. Viral trafficking in HeLa cells was impaired by treatment with TET or CEP. Representative fluorescence images indicated the time-dependent accumulation of EV71 viral particles in HeLa cells treated with TET or CEP, both at 10 μM compared to the control (DMSO) over a period of 120 min. At 15, 30, 45, 60, 90, and 120 min p.i., the HeLa cells were fixed and for the viral capsid protein VP1 to visualize the viral signals (green puncta). The nuclei were stained with DAPI (blue). Scale bar represent 20 μm.

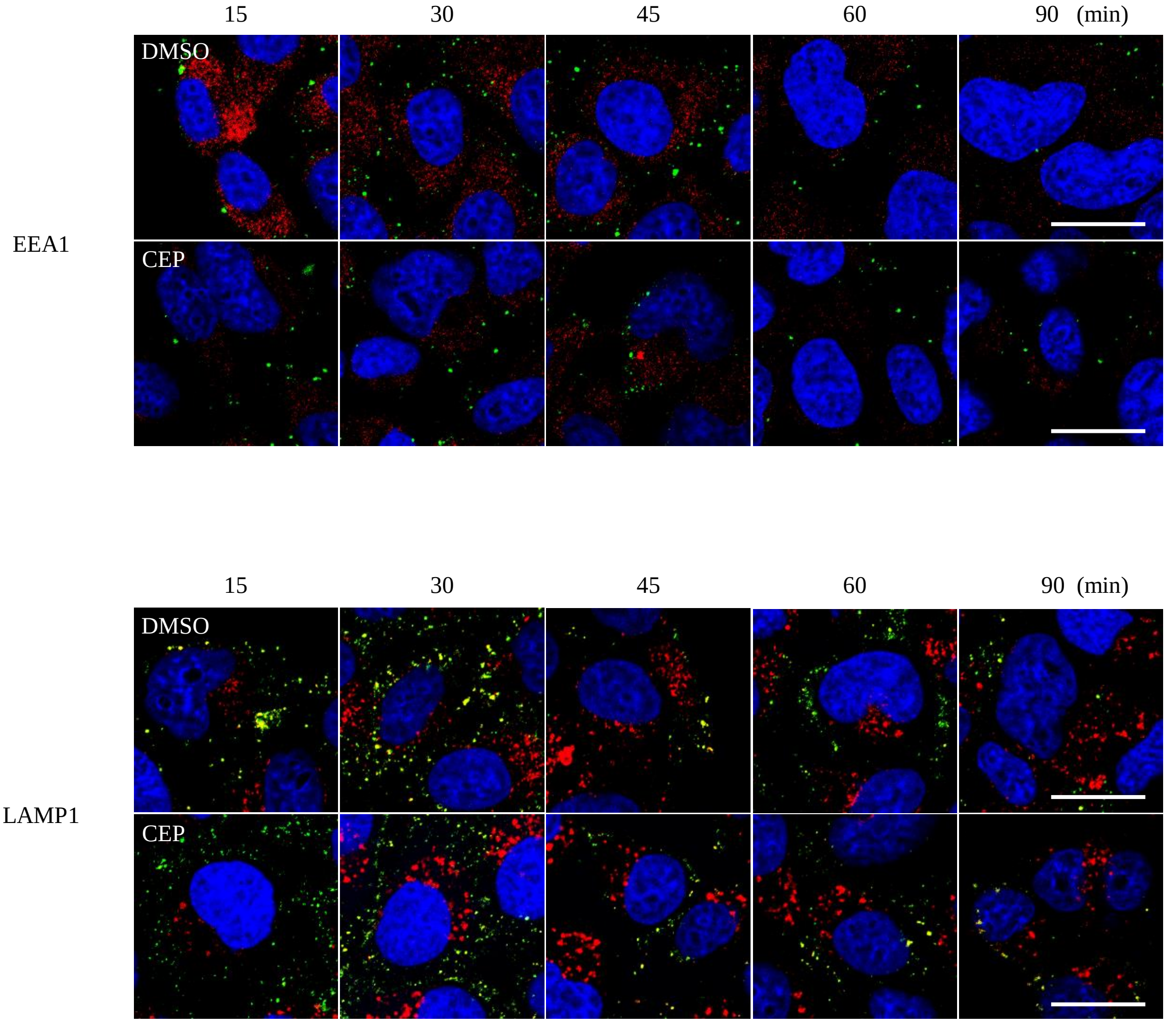

Figure S3. The trafficking of EV71 particles along the endolysosomal pathway was delayed by CEP. HeLa cells were infected with EV71 at an MOI of 300 at 4 °C for 1 h to allow for viral adsorption. After this period, the cells were washed and then transferred to 37 °C, where they were treated with either a vehicle control (DMSO) or 10 µM CEP. At specified time points following the temperature shift (15, 30, 45, 60, and 90 min), the cells were fixed, permeabilized, and triple-stained. The staining involved using a mouse anti-VP2 antibody with an Alexa Fluor 488 secondary antibody (which indicates EV71 virions in green), as well as Alexa Fluor 546-conjugated antibodies against either EEA1 (early endosomes, shown in red in the upper panels) or LAMP1 (lysosomes, shown in red in the lower panels). DAPI was used to stain nuclei, appearing in blue. Areas where the green and red signals overlap appear yellow, indicating the colocalization of virions with the respective organelle markers. The micrographs presented are representative images used for the quantitative colocalization analyses (Fig. 4D and 4E). Scale bar represent 20 µm.

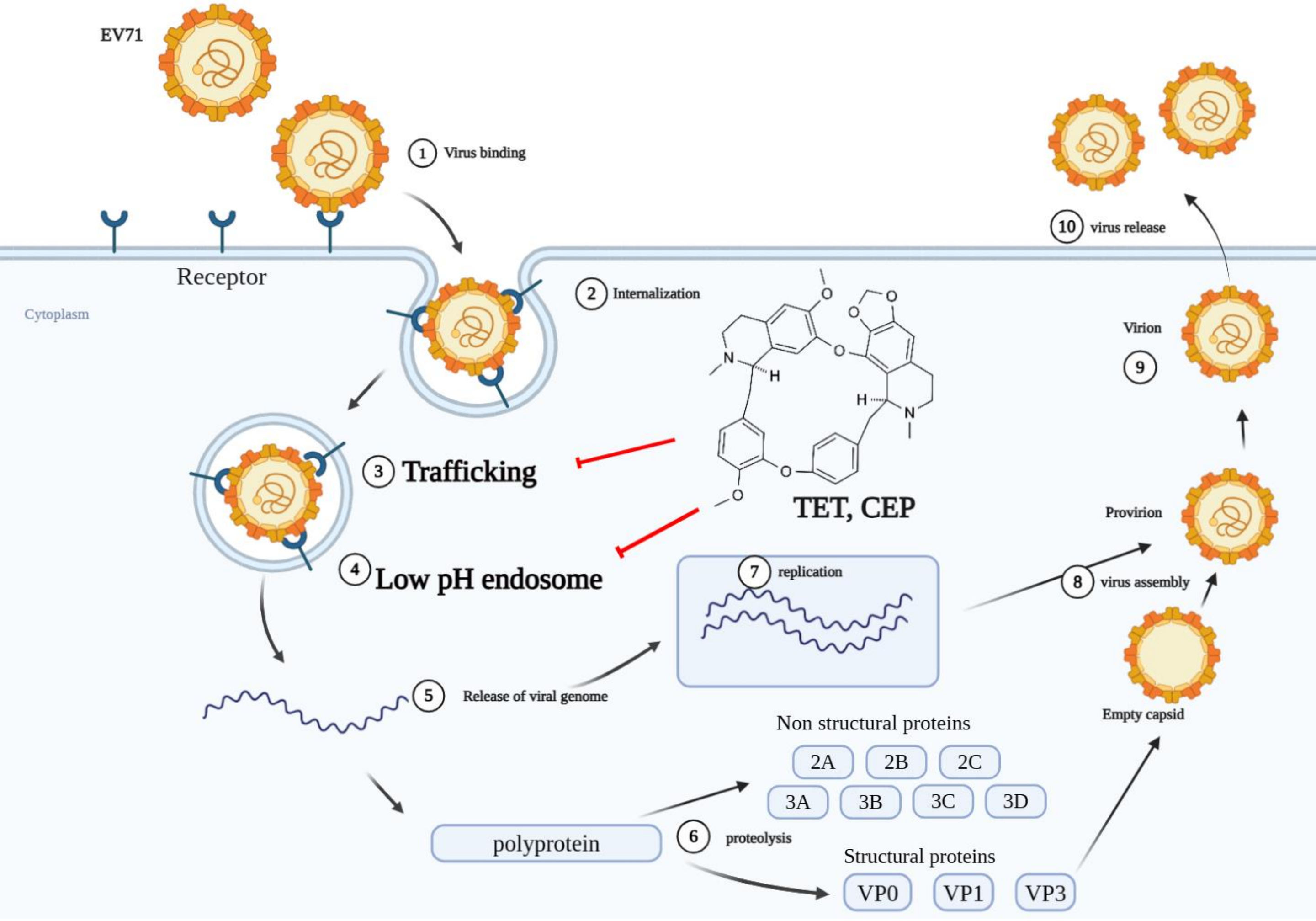

Figure S4. Schematic representation of the antiviral mechanisms employed by TET and CEP against EV71. Throughout the viral infection cycle, encompassing steps 1 to 10, TET and CEP exhibit antiviral activity that specifically targets viral trafficking (Step 3, Fig. 4) and the progressive acidification (Step 4, Fig. 5) along the endolysosomal pathway.

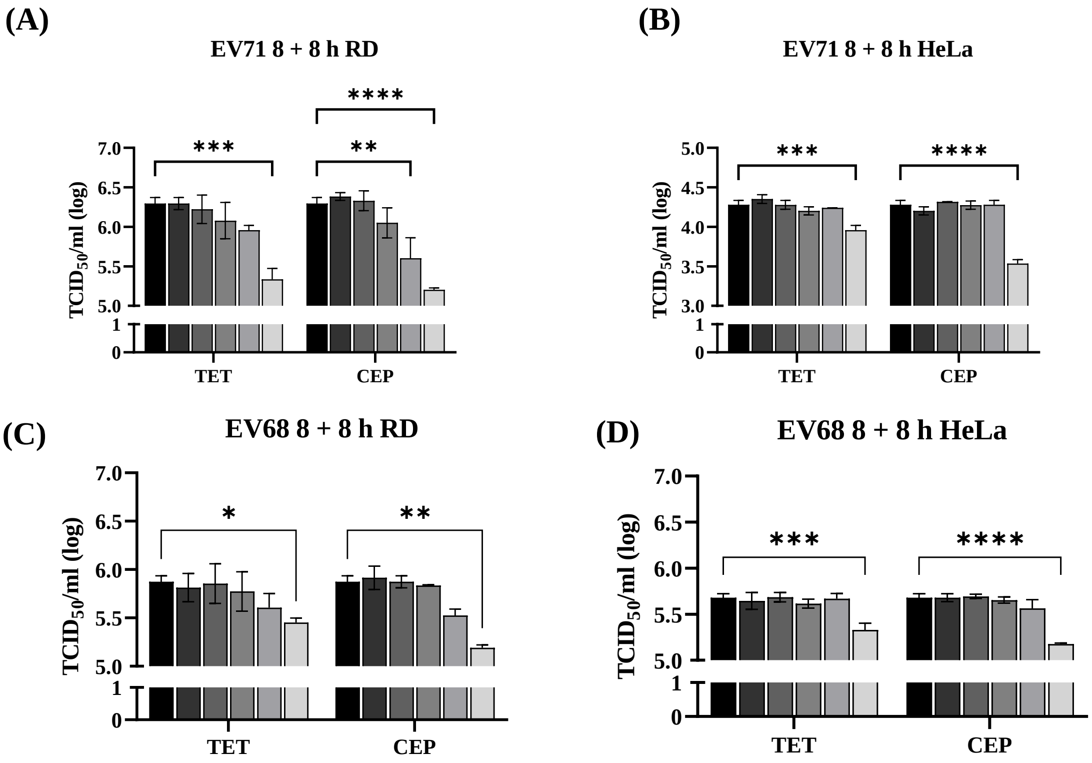

Fig. S5. Mid-course replenishment demonstrates comparable antiviral potency of CEP to a single dose treatment over a 16-h infection period. RD (A, C) or HeLa cells (B, D) were infected with EV71 (A, B) or EV68 at an MOI of 0.1 and treated with CEP or TET at the indicated concentrations. Compounds were first added at 0 h p.i. and refreshed with the same concentration at 8 h p.i. (denoted as 8 + 8 h). Total viral titers were quantified using the $TCID_{50}$ assay. The graphs present mean values along with standard deviations (n = 3). The $TCID_{50}$ data were analyzed using ANOVA, with significance indicated as ****P < 0.0001, ***P < 0.001, **P < 0.01, *P < 0.05.

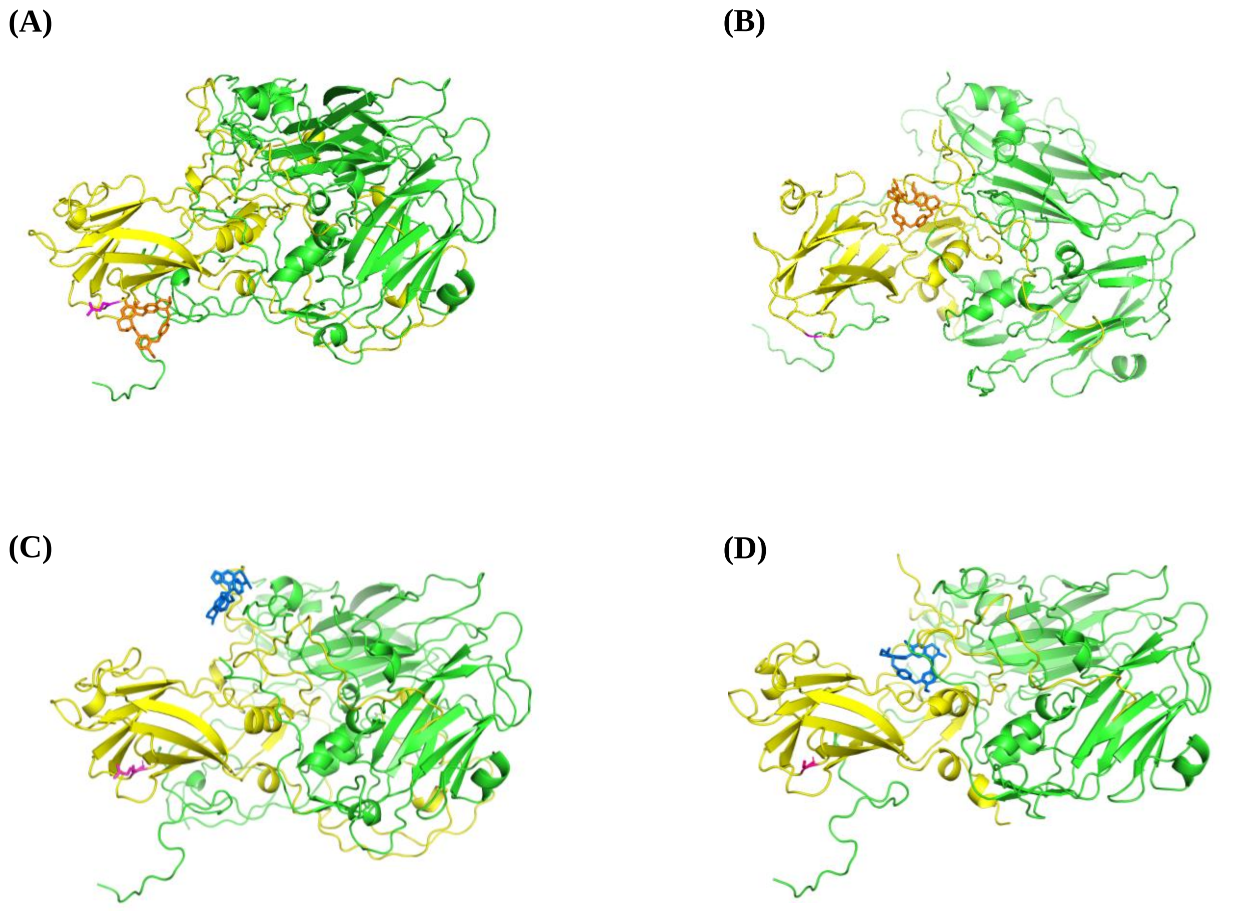

Figure S6. Docking models for FAN and CEP interacting with either wild-type E145 or mutant E145G of EV71 VP1. The interactions between FAN or CEP and either the wild-type E145 (protein data bank (PDB) ID: 3VBS) or the mutant E145G (PDB ID: 4GMP) were modeled using AutoDock 4.2 software, as referenced in PMID: 37193133. The 3D structures of FAN and CEP were obtained from NCBI PubChem. (A) FAN docked to VP1 E145. (B) FAN docked to VP1 E145G. (C) CEP docked to VP1 E145, and (D) CEP docked to VP1 E145G. In the models, VP1 is depicted in yellow, while VP2 through VP4 are shown in green. Residue 145 is highlighted in magenta, CEP poses are illustrated in blue, and FAN poses are displayed in orange. The data indicates that FAN binds strongly to VP1 E145 with a binding energy of -6.92 kcal mol^{-1}; however, it is unable to bind to VP1 E145G. In contrast, CEP does not bind to either VP1 E145 or VP1 E145G.
